# Resolving kangaroo phylogeny and overcoming retrotransposon ascertainment bias

**DOI:** 10.1038/s41598-017-16148-0

**Published:** 2017-12-01

**Authors:** William G. Dodt, Susanne Gallus, Matthew J. Phillips, Maria A. Nilsson

**Affiliations:** 1 School of Earth, Environmental and Biological Sciences, Queensland University of Technology (QUT), 2 George Street, Brisbane, Australia; 2 Senckenberg Biodiversity and Climate Research Centre (BiK-F) Frankfurt, Senckenberg Gesellschaft fuer Naturforschung, Senckenberganlage 25, Frankfurt am Main, Germany

## Abstract

Reconstructing phylogeny from retrotransposon insertions is often limited by access to only a single reference genome, whereby support for clades that do not include the reference taxon cannot be directly observed. Here we have developed a new statistical framework that accounts for this ascertainment bias, allowing us to employ phylogenetically powerful retrotransposon markers to explore the radiation of the largest living marsupials, the kangaroos and wallabies of the genera *Macropus* and *Wallabia*. An exhaustive *in silico* screening of the tammar wallaby (*Macropus eugenii*) reference genome followed by experimental screening revealed 29 phylogenetically informative retrotransposon markers belonging to a family of endogenous retroviruses. We identified robust support for the enigmatic swamp wallaby (*Wallabia bicolor*) falling within a paraphyletic genus, *Macropus*. Our statistical approach provides a means to test for incomplete lineage sorting and introgression/hybridization in the presence of the ascertainment bias. Using retrotransposons as “molecular fossils”, we reveal one of the most complex patterns of hemiplasy yet identified, during the rapid diversification of kangaroos and wallabies. Ancestral state reconstruction incorporating the new retrotransposon phylogenetic information reveals multiple independent ecological shifts among kangaroos into more open habitats, coinciding with the Pliocene onset of increased aridification in Australia from ~3.6 million years ago.

## Introduction

The genus *Macropus* includes kangaroos, wallaroos and wallabies, which are herbivorous, and occupy a wide range of terrestrial habitats throughout Australia, parts of New Guinea and several surrounding islands^1^. The 13 species are currently grouped into three subgenera - the predominantly mesic members of *M*. (*Macropus*) and *M*. (*Notamacropus*), as well as the more arid adapted members of *M*. (*Osphranter*)^2^. The evolutionary relationships among these subgenera and their relationship to the swamp wallaby (*Wallabia bicolor*) have remained contentious^3–5^. Studies based on maternally inherited mitochondrial DNA (mtDNA) have favoured a sister relationship between *Wallabia* and the genus *Macropus* ^4,6^. This is broadly in agreement with morphological studies, which have placed *Wallabia* outside of *Macropus* ^7,8^. However, analysis of five concatenated nuclear genes provided moderate support for *Wallabia bicolor* being nested inside *Macropus* ^3^. Conversely, the traditional placement of the black-gloved wallaby (*Macropus irma*) within *M*. (*Notamacropus*) is supported by nuclear DNA^3^, whereas analysis of mtDNA instead placed *Macropus irma* within *M*. (*Osphranter*)^4^. The sister group to *Macropus* and *Wallabia* also remains unclear. Morphological characters arguably favour the nail tail wallabies (*Onychogalea*) as the sister group to *Macropus* and *Wallabia* ^5,7,8^, while five concatenated nuclear loci weakly favour the hare wallabies (*Lagorchestes*)^3^, and mtDNA analyses remain uncertain^9^.

*Macropus* and *Wallabia* stem from within a broader adaptive radiation of macropodid genera (also including *Lagorchestes*, *Onychogalea*, and *Setonix*) that took place during the Late Miocene, a period of gradual cooling, drying and opening of forests across Australia^3,8,10,11^. According to previous molecular dating estimates, the divergence of all three *Macropus* subgenera and *Wallabia* took place over a period of 1–2 million years^3,4^. This rapid radiation is consistently associated with low statistical support among both nuclear and mitochondrial DNA for relationships among the three subgenera of *Macropus*. This uncertainty precludes confident inference of whether habitat expansion into semi-arid grasslands and grazing specializations (see ref.^12^) evolved early in macropodids or later, independently in the *Macropus* subgenera, *M*. (*Osphranter*) and *M*. (*Macropus*), and also in *Onychogalea*.

Next generation DNA sequencing and the increasing availability of complete nuclear genomes have allowed phylogenetic relationships to be investigated using novel methods that utilize genome level characters^13^. Here we employ a genome-wide retrotransposon presence/absence analysis, in an attempt to resolve the evolutionary history of the genera *Macropus* and *Wallabia*. Retrotransposons have a number of advantages over traditional sequence based phylogenetic reconstruction. Most notably, retrotransposons are a virtually homoplasy-free marker system^13–15^, due to near-random insertion across the genome providing an almost unlimited size of character space. Traditional DNA sequence-based methods, which are more prone to homoplasy within loci, can obscure gene tree affinities. Furthermore, retrotransposon analyses utilize a relatively simple and unambiguous parsimony approach^13^, unlike sequence-based methods that require complex models of molecular evolution.

**Figure 1:**
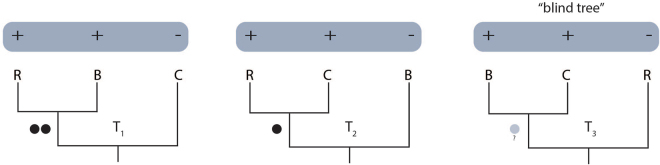
Hypothetical example illustrating the “blind” tree scenario that occurs when only a single reference genome is available. Initial *in silico* screening of the reference genome (R) will have identified the markers for experimental screening across taxa, such that insertions will only be observable in clades that include taxon R. In T_1_ and T_2_, the reference genome (species R) groups with species B and C respectively. However T_3_ is referred to as the “blind” tree since any retrotransposon insertion supporting species B and C grouping together will be unobservable. Black circles represent observable retrotransposon markers supporting topologies (T_1_ and T_2_); the grey circle represents potential retrotransposon markers that are unobservable in this scenario.

Hypothesis testing with retrotransposons has typically assumed equal prior probability of identifying markers supporting each of the three bifurcating topologies that could be resolved from a phylogenetic trichotomy^16^. However, few reference genomes of closely related taxa are available for screening retrotransposon insertions, which results in an ascertainment bias^17^. Specifically, markers that support any grouping that does not include a reference taxon are unlikely to be observed, such that the retrotransposon method is “blind” to some trees (Fig. 1)^17,18^. This is a particularly critical problem for retrotransposon studies based on a single reference genome e.g. refs^19–21^. Avoiding an ascertainment bias requires n-1 reference genomes for each set of n taxa within the phylogeny of interest. Thus with only four distantly related genomes published so far (opossum, Tammar wallaby, Tasmanian devil and koala)^22–25^, marsupials (and most other taxa) will continue to be susceptible to the retrotransposon ascertainment bias for the foreseeable future. This highlights the importance of developing analytical methods to overcome genome scarcity, which will in turn, allow researchers to confidently infer phylogenies from retrotransposon markers.

Kuritzin *et al*.^18^ provided the first step in accommodating the single reference genome ascertainment bias by amending the previously published P-value calculations of Waddell *et al*.^16^ for hypothesis testing with retrotransposons (Materials and Methods). To meet a significance level P = 0.05, a minimum of three unopposed markers (P = 0.0370) are sufficient when the ascertainment bias does not need to be considered. However, five unopposed binary markers are required (P = 0.0370) with the ascertainment bias. Kuritzin *et al*.^18^ suggested that when only a single reference genome is available, the ascertainment bias renders phylogenetic resolution impossible, due to the “blind” tree. We present three arguments that can be used to reject the “blind” tree and confirm the phylogeny or alternatively, to show that additional reference genomes will be required. These arguments include consideration of *a priori* evidence, discussed on a case by case basis, and two tests for whether the observed markers could be hemiplasic instead of reflecting species relationships. We define hemiplasy inclusively, to include its original usage for incomplete lineage sorting (ILS)^26^, as well as introgression, which often cannot be distinguished from ILS for individual loci^4^. The statistical tests are independent of any *a priori* evidence and allow us to infer whether the observed markers could derive from (i) ILS, which is expected to distribute markers symmetrically between the two non-species trees or (ii) introgression/hybridisation, which influences the ratio of observed markers on successive branches (Materials and Methods). We employ this statistical framework to account for the ascertainment bias that arises when relying on only the *Macropus eugenii* reference genome^27^ to extract phylogenetically informative retrotransposons. We present the first retrotransposon-based phylogeny of kangaroos and wallabies (Family Macropodidae), and trace their adaptive diversification over the past 10 million years.

## Materials and Methods

### Assessment of retrotransposon activity

Initial selection of potentially phylogenetically informative markers was carried out using the reference genome assembly of *Macropus eugenii* (version macEug2, http://ucsc.genome.edu). The *M*. *eugenii* genome assembly was used to extract single-copy introns and/or intergenic regions containing retrotransposons (Supplementary Information). Resulting sequence alignments were repeat masked using either RepeatMasker^28^ or CENSOR, http://www.girinst.org/censor/index.php ^29^ to identify the position of repeat elements within the sequence and facilitate primer design. Primers were designed to flank repetitive elements approximately 250–400 nt either side of the retrotransposon (Supplementary Table S1). Primer specificity for single copy regions was tested in silico for the *M*. *eugenii* genome.

### Experimental verification

DNA extractions (Supplementary Table S2) were performed using standard phenol chloroform extraction^30^ or the gDNA extraction kit (Promega). Conditions for PCR screening are given in the Supplementary Information. The amplicon size difference between species for each primer pair (visualized by gel-electrophoresis) was used to predict informative phylogenetic markers. The selected markers were verified by Sanger sequencing to validate the presence of Kangaroo Endogenous RetroViral Element-1 (KERV-1) insertions and target site duplications^27^. Representatives from multiple taxa following insertion and one or more taxa without the insertion were Sanger sequenced to verify PCR patterns for all markers. For closely related species within lineages, Sanger sequences and gel electrophoresis presence of a ‘filled site’, exemplified by a ~400 nt larger PCR product, was used to establish the phylogenetic position of the marker (Supplementary Material). When direct sequencing was problematic, PCR products were ligated into the pDrive plasmid (Qiagen) prior to sequencing. All sequences were visually inspected and aligned in Se-AL 2.0^31^.

### Calculation of retrotransposon phylogenetic support values

**Figure 2:**
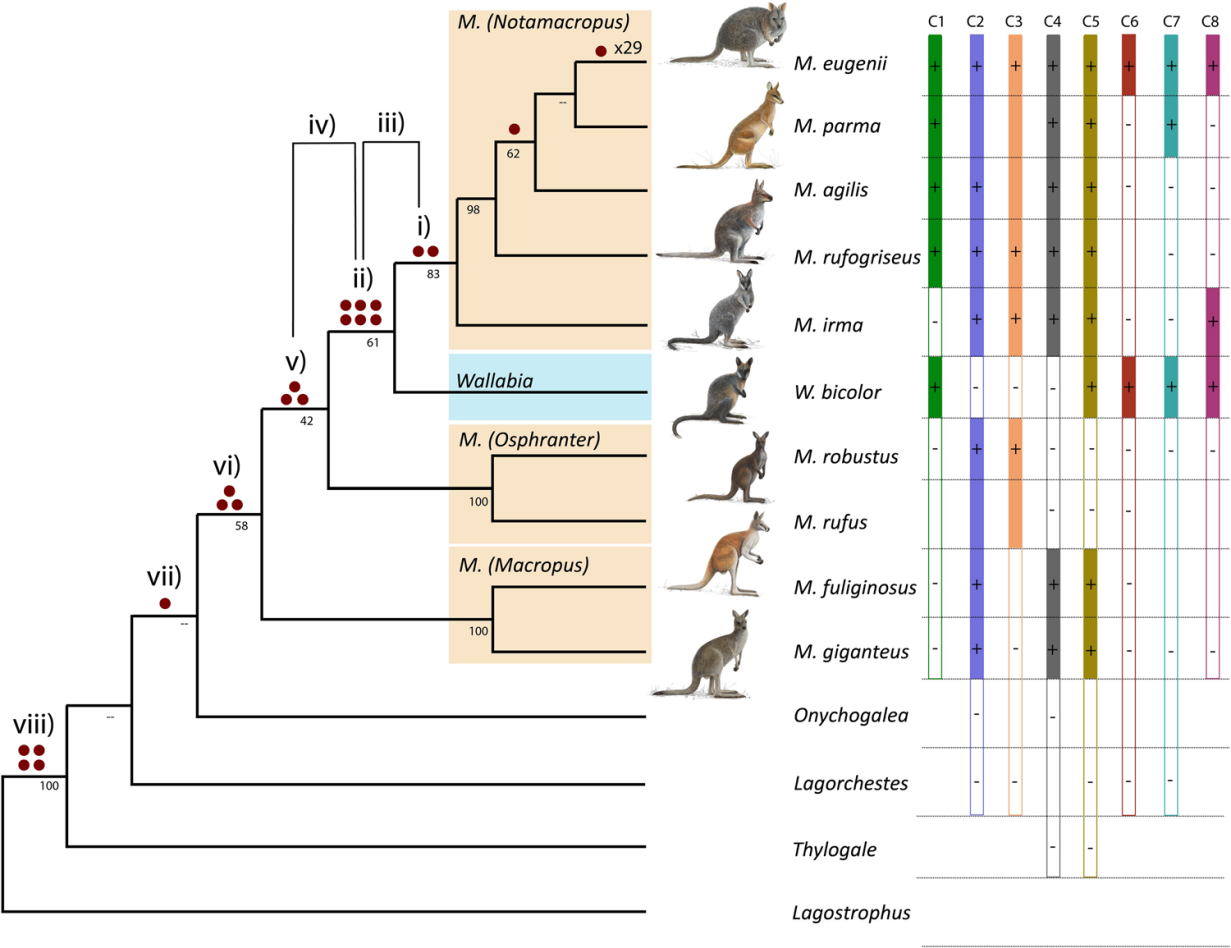
Kangaroo and wallaby maximum parsimony phylogeny inferred from retrotransposon data. Dark red circles represent retrotransposon markers. Clades for which there is no retrotransposon information (shown here without red dots) were then resolved in agreement with phylogenetic analyses of both nuclear and mitochondrial DNA sequences^3,4,39^. ML support values are obtained from an independent analysis^3^ that utilized nuclear genes, for comparison with the retrotransposon markers. Shaded rectangles represent the genus *Wallabia* (light blue) and the *Macropus* subgenera (orange). Coloured vertical bars (C1 – C8) each represent a retrotransposon marker that conflicts with the majority based on parsimony inference, and indicate alternative groupings of taxa (e.g. conflict bar C1 supports a grouping of *M*. *eugenii* + *M*. *agilis* + *M*. *parma* + *M*. *rufogriseus* + *W*. *bicolor* to the exclusion of *M*. *irma* and other macropods). Identified presences and absences are respectively marked (+) and (−) or unmarked if data is missing. Outgroup species are *Onychogalea unguifera*, *Lagorchestes hirsutus*, *Thylogale thetis* and *Lagostrophus fasciatus*. Marker indexes refer to Table 2, and are numbered i) to viii), with iii) and iv) both summing markers along two branches. The location of the markers is based on Sanger sequencing and scoring filled and empty sites from PCR amplification patterns (Supplementary Table S3, Figure S3a,b). Kangaroo images by Jon Baldur Hlidberg.

The sequence alignments and PCR gel-electrophoresis patterns were used to catalogue the presence/absence of phylogenetically informative markers (Supplementary Table S3), which are plotted on the tree in Fig. 2. Our strategy was to establish the branch on which insertion occurred, focusing on sampling *Macropus* (and *Wallabia*), and also showing that the marker is not present on at least two (ideally successively) deeper lineages. The only less stringent exceptions are K136, which was thus not employed for hypothesis testing (but could be included for parsimony analysis), and K107, which was absent only in the deepest macropod. Several untested markers in *M*. *parma* are due to limited DNA availability, but multiple other members of *M*. (*Notamacropus*) were tested. *Wallabia* and at least one of the closely related members of *M*. (*Osphranter*) and *M*. (*Macropus*) were sampled for all markers pertaining to relationships within Macropodinae.

P-values for each branch were calculated for the retrotransposon data using binomial probability based on a similar statistical approach to that described by Kuritzin *et al*.^18^, which updates the statistics of Waddell *et al*.^16^ (Table 1). The P-values are based on the probability of random allocation of markers to the three alternative bifurcating tree hypotheses that can be resolved from a trichotomy. Consider Fig. 1, where 2, 1 and 0 markers respectively support the three topologies; T_1_ ((R, B), C), T_2_ ((R, C), B), and T_3_ ((B, C), R), where taxon R is the reference genome. This gives a retrotransposon count of [2 1 0]. The exact cumulative binomial probability (P_B_) for T_1_ being supported by at least two of three markers, each with probability 1/3, is P_B_ = 0.2593.

**Table 1:** Cumulative P-values for testing prior tree hypothesis T_1_ on retrotransposon counts^18^ amended from^16.^ Additional P-values can be calculated at http://retrogenomics.uni-muenster.de:3838/KKSC_significance_test/.

Multi-directional searches: markers are equally identifiable for the three trees				One-directional searches: markers for the third tree are unidentifiable			
Insertion count	P value^a^	Insertion count	P value^a^	Insertion count	P_B_ value^b^	Insertion count	P_B_ value^b^
[1 0 0]	0.3333	[5 1 0]	0.0178	[1 0 X]	0.5000	[5 1 X]	0.1094
[1 1 0]	0.5556	[5 2 0]	0.0453	[1 1 X]	0.7500	[5 2 X]	0.2266
[2 0 0]	0.1111	[6 0 0]	0.0014	[2 0 X]	0.2500	[6 0 X]	0.0156
[2 1 0]	0.2593	[6 1 0]	0.0069	[2 1 X]	0.5000	[6 1 X]	0.0625
[2 2 0]	0.4074	[6 2 0]	0.0197	[2 2 X]	0.6875	[6 2 X]	0.1445
[3 0 0]	0.0370	[7 0 0]	0.0005	[3 0 X]	0.1250	[7 0 X]	0.0078
[3 1 0]	0.1111	[7 1 0]	0.0026	[3 1 X]	0.3125	[7 1 X]	0.0352
[3 2 0]	0.2099	[7 2 0]	0.0083	[3 2 X]	0.5000	[7 2 X]	0.0898
[4 0 0]	0.0123	[8 0 0]	0.0002	[4 0 X]	0.0625	[8 0 X]	0.0039
[4 1 0]	0.0453	[8 1 0]	0.0010	[4 1 X]	0.1875	[8 1 X]	0.0195
[4 2 0]	0.1001	[8 2 0]	0.0034	[4 2 X]	0.3438	[8 2 X]	0.0547
[5 0 0]	0.0041	[9 0 0]	0.00005	[5 0 X]	0.0313	[9 0 X]	0.0020

^a^For cases when all markers are observable (P); ^b^Revised values to accommodate the single reference genome bias (P_B_), excluding markers supporting T_3_.

The ascertainment bias resulting from using a single reference genome requires further amendment, because markers supporting the clade that does not include the reference genome are not observable. Hence, we refer to T_3_ ((B, C), R) as the “blind” tree. The retrotransposon count reduces to [2 1 X], with X indicating unknown status. Hypothesis testing becomes binary, and [2 1 X] is among 2^3^ = 8 permutations for three markers ([3 0 X], [0 3 X], and three permutations each for [2 1 X] and [1 2 X]). Half of these permutations ([3 0 X] and the three permutations for [2 1 X]) are at least as favourable for T_1_ as is the observed count [2 1 X]. Hence, when acknowledging that markers for the “blind” T_3_ are unobservable, the exact cumulative binomial probability for tree 1 being supported by at least two of three markers, each with probability ½, increases to P_B_ = 0.5.

The binomial probability calculations with all markers being observable are identical to Kuritzin *et al*.’s^18^ “multi-directional KKSC test” and binomial probability calculations with one clade being “blind” to insertions are identical to the “one-directional KKSC test”. Given this equivalence with cumulative binomial probability, we will refer to these binomial probability tests as KKSC (P_B_) tests.

### Derivation of arguments for overcoming the single reference genome ascertainment bias

A significant KKSC (P_B_) test rejects the null hypothesis that there is no difference in support for the two observable trees. To reject the “blind” tree, two further hypotheses need to be rejected. These are, *H*_ILS_: that the “blind” tree is the species tree and markers supporting the observable trees result from ILS, and *H*_Introgression_: that the “blind” tree is the species tree and markers supporting the observable trees result from introgression/hybridization.

We employ an ILS symmetry argument to test *H*_ILS_. Theory^18,32,33^ and observed patterns^34,35^ show that ILS will distribute markers that conflict with the species tree roughly symmetrically among the two non-species tree alternatives (the two observable trees, if the “blind” tree is the species tree). The multi-directional KKSC-hybridization test of Kuritzin *et al*.^18^ (http://retrogenomics.uni-muenster.de:3838/KKSC_significance_test) tests whether ILS alone can explain the difference in the number of markers supporting the two clade hypotheses with the fewest insertions. Our ILS test is a special case of the KKSC-hybridization test, in which the hypothesis specifies that the “blind” tree X is the species tree. Therefore X can take any value ≥ the number of markers supporting the favoured observable clades. Conveniently, under this condition the multi-directional KKSC-hybridization test (and ILS test) is independent of X.

**Figure 3:**
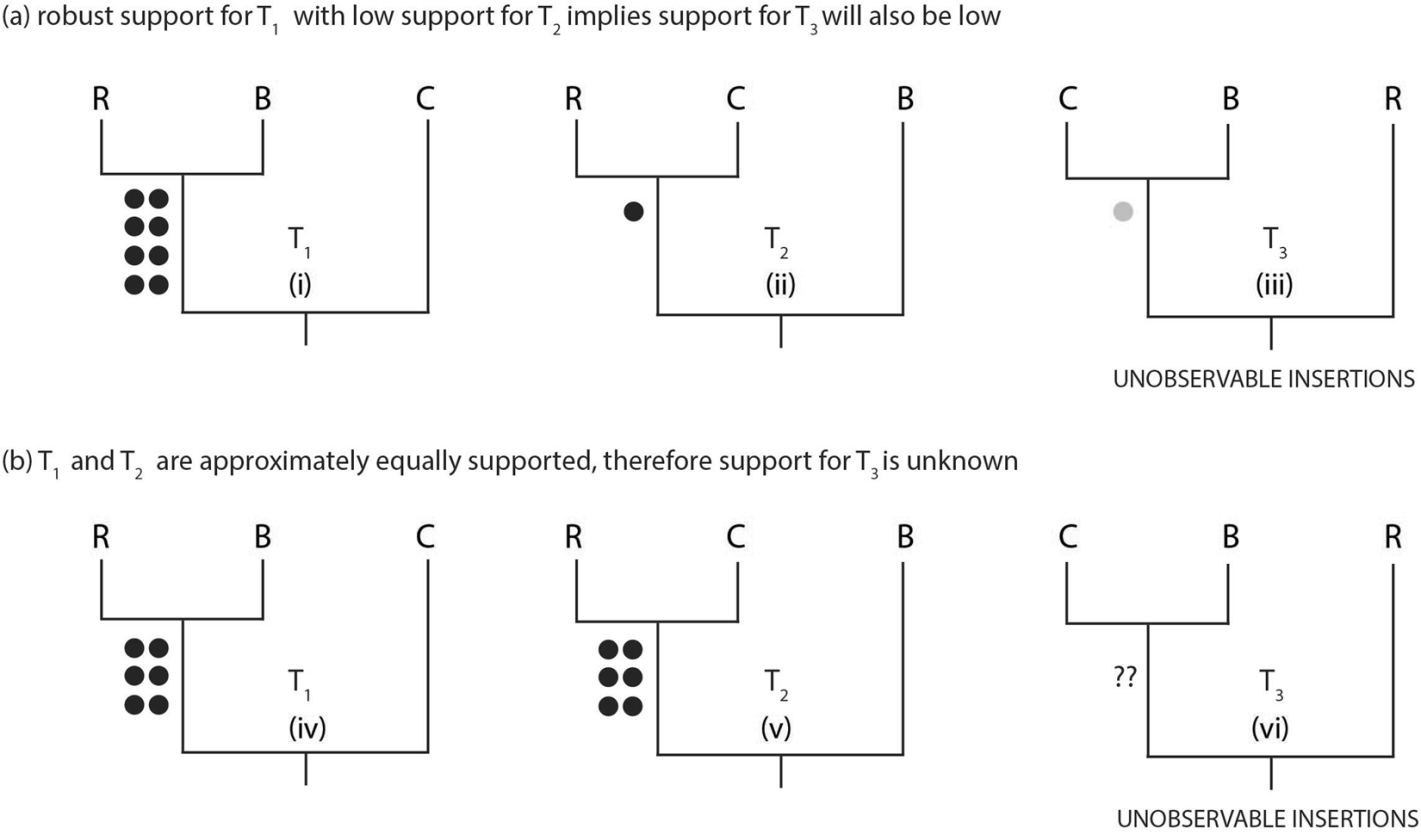
ILS symmetry test. Hypothetical scenario illustrating the ILS symmetry test for accepting or rejecting the “blind” tree, under the assumption of ILS. There are three possible topologies (T_1_–T_3_) for clades R, B and C. Different numbers of retrotransposon markers are observed (black dots) or principally unobservable (grey dot), when genome data is only available for the reference genome, R. Generally, the species tree is expected to have the highest number of markers, while ILS is expected to distribute insertions approximately evenly between the two alternative (non-species tree) groupings. Under ILS, in (**a**) if T_1_ (i) has numerous markers, and T_2_ (ii) has few markers, then the “blind” T_3_ (iii) is also expected to have few markers (maintaining ILS symmetry), and T_1_ can be inferred to be the species tree. If few insertions occurred along the (R, B) stem lineage of the species tree, that topology may not be supported by significantly more insertions than ILS distributes to the non-species tree alternatives. Hence, in (**b**) if T_1_ (iv) and T_2_ (v) are supported by a similar number of markers, symmetry in the number of deep coalescences between the two non-species trees could arise in two ways. T_3_ (vi) could be the species tree and potentially supported by a larger number of unobserved markers (ILS symmetry between T_1_ and T_2_) or either T_1_ or T_2_ could be the species tree with a similar number of markers supporting (C, B) and ILS symmetry maintained between T_2_ and T_3_ or T_1_ and T_3_, respectively.

In Fig. 3 the “blind” tree, T_3_ (iii) is ((B, C), R), where R is the reference genome. Significant disparity in the number of markers supporting trees T_1_ (i) and T_2_ (ii) allows us to reject the hypothesis that the observed markers resulted from ILS, and hence, reject *H*_ILS_. The ILS (and KKSC-hybridization) test is the same binomial probability test as for KKSC (P_B_), except P_ILS_ is two-tailed, because the disparity could be T_1_ > T_2_ or T_1_ < T_2_. ILS tests for the hypothetical examples in Fig. 3 reject the “blind” T_3_ (iii) for (a) [8 1 X] P_ILS_ = 0.0391, but cannot reject the “blind” T_3_ (vi) for (b) [6 6 X] P_ILS_ = 1.0.

Rejecting *H*_ILS_ still leaves the possibility that the “blind” tree is the species tree – if the observable markers result from introgression/hybridization (*H*_Introgression_). We have developed a test for hybridization/introgression that considers the number of markers identified on successive branches along the stem lineage of the reference taxon. This “insertion ratio test” exploits the biological expectation that the proportion of insertions that introgression shares is governed by the proportion of the genome shared. If the “blind” tree is the species tree, these introgressed markers will instead appear to support a non-species tree that includes the reference taxon.

**Figure 4:**
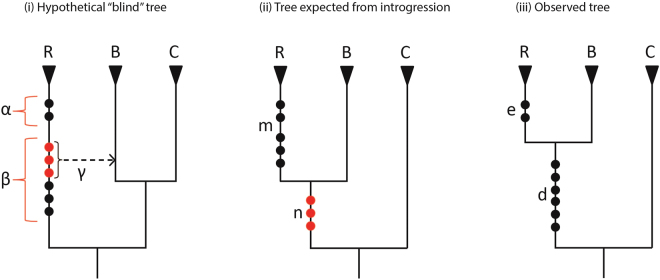
Insertion ratio test. Hypothetical scenario illustrating the ‘insertion ratio test’ for accepting or rejecting the “blind” tree under the assumption of introgression/hybridization. (i) Hypothetical “blind” tree with α and β representing insertions respectively occurring after and before an introgression event between the stem lineages of species R (reference genome) and species B. The proportion of the genome shared and retained between species R and B is γ. (ii) The expected tree under this hypothetical introgression scenario, with γ = 0.5. The expected number of insertions from introgression supporting (R + B) is *n* = β γ and the expected number of unshared insertions (and so, unique to R) is *m* = α + β(1-γ). (iii) The experimentally identified “observed tree” with *d* markers supporting R + B and *e* markers unique to the branch leading to the reference genome, R. If *d* is significantly higher than *n* under binomial probability, we reject the hypothesis that introgression/hybridization can explain the level of support for the observed tree. A maximum value for *n* can be estimated as *N* = γ(*d* + *e*), see Materials and Methods.

A hypothetical example of introgression is illustrated in Fig. 4. We start by assuming the “blind” tree (i) is the species tree (grouping taxa B + C). The parameters α and β are the respective numbers of markers that inserted before and after a proportion (γ) of the reference genome (R) was shared and remains with taxon B. This genetic sharing favours the observed tree (ii), grouping the reference (R) with taxon B. The expected number of introgressed markers supporting this “incorrect” tree is *n* = βγ. The number of markers expected along the stem lineage of R is *m* = α + β(1 − γ), where the term β(1 − γ) is the number of markers along the lineage leading to R that inserted before the introgression event, but are in the portion of the genome not shared with taxon B.

The insertion ratio test rejects the hypothesis that the observed tree (Fig. 4iii) derives from introgression/hybridization, if the number of markers (*d*) supporting (R + B), is significantly greater than introgression is expected to contribute, denoted *n* in (ii). Unfortunately, we cannot know the true value of *n* (recall that *n* = βγ), because the number of markers (β and α) in (i) and the shared proportion of the genome shared (γ) are all unknown. However, we note that *n* is maximized when α = 0 and γ is high, e.g. 0.5 would be an extreme value for γ. Then knowing only the observed tree (iii), the maximum value of *n* (which we denote *N*) is the proportion of the genome shared (γ), multiplied by the number of markers potentially available in stem-R for sharing (*d* + *e* = 8). In this scenario *N* = γ(*d* + *e*) = 4, which being a maximum value, provides a conservative insertion ratio test.

The insertion ratio test is denoted P_R50_ when γ = 0.5, and is expressed in the form (*d*,*e*) (see Fig. 4iii) as a standard cumulative binomial probability test, for which the number of “trials” (markers) is *d* + *e*, the number of “successes” (markers shared) is *d*, and the “expected probability of success” for each trial reduces to γ. Returning to the observable tree in Fig. 4iii, the clade (R + B) is supported by *d* = 6 markers and the next (shallower) clade that includes the reference taxon is supported by *e* = 2 markers. With this observed insertion ratio count (6,2) in Fig. 4iii, P_R50_ = 0.1445. Essentially, the observed support (*d* = 6) is not significantly higher than expected under 50% introgression (*N* = 4). Hence, we cannot reject *H*_Introgression_: “blind” species tree with 50% introgression/hybridization contributing the markers supporting (R + B).

Setting the shared and retained proportion of the genome (γ) to 0.5 is an extreme scenario, such as for homoploid hybrid species derived purely from F1 hybrid ancestors. Thus, P_R50_ is very conservative. On both morphological and population genetic evidence there is only support for lower level introgression, even among closely related kangaroos^4,36^, and so we also present a more realistic scenario for the insertion ratio test, with γ = 0.2 (P_R20_). In the Fig. 4(iii) example, this reduces *N* to 0.2 × (6 + 2) = 1.6, and for (6,2), P_R20_ = 0.0012. If the ILS test and the insertion ratio test respectively reject *H*_ILS_ and *H*_Introgression_, then at least some markers supporting the observed tree derive from shared species tree ancestry, and we can reject the “blind” tree.

Molecular dating and ancestral habitat reconstruction was carried out with MrBayes 3.2.6^37^. The analyses employed the five nuclear gene (Rag1, BRCA1, vWF, IRBP, ApoB) data matrix of Meredith *et al*.^3^ for 31 macropods (Macropodiformes), and seven outgroup species, with temporal calibration from six fossil-based priors (Supplementary Information).

Ethics statement: All specimens were already deceased at the time of sampling (ie. Roadkill, animal shelters, wildlife sanctuaries and zoos). Tissue use is covered by QUT ethics approval confirmation number 1400000559. No further experimental ethics requirements are necessary, given the nature of this research.

### Data Availability

All alignments are available in fasta-format as Supplementary material. All sequences have been deposited into the European Nucleotide Archive under the accession numbers: LT598171 -LT598445.

## Results

### Activity of an endogenous retrovirus during the evolution of *Macropus*

An exhaustive *in silico* screen of the reference genome, *Macropus eugenii*, identified three prevalent retrotransposon types, LINE (LINE1), SINE (WALLSI2) and ERV (KERV-1 deposited in Repbase as MERVK1) elements. 38 LINE1 and seven WALLSI2 loci were screened experimentally, but these elements showed no phylogenetic activity for the investigated branches as the markers were present in all tested species. We experimentally screened 83 KERV-1 (MERVK1) loci across 16 macropodiform species, covering the major clades over ~25 million years of evolution, and identified 29 phylogenetically informative solo-LTRs (Fig. 2 and Supplementary Table S4). Each KERV-1 insertion is flanked by 5–6 nt long target site duplications without a common motif (Supplementary Table S4). Many of the examined ERVs appear to have diagnostic sequence changes (e.g. 40 nt deletions) separating them from previously published KERV-1 elements, suggesting that the number of currently described KERV-1 sub-families has been underestimated. Additional screening was carried out in six *Macropus eugenii* individuals to investigate polymorphic retrotransposon markers (Supplementary Table S5), which suggest recent or ongoing retrotransposition. To reduce the single reference genome ascertainment bias, 33 introns that lacked retrotransposon insertions in *Macropus eugenii* were also experimentally screened in additional species, but yielded no novel markers. The 29 phylogenetically informative KERV-1 markers are shown in Fig. 2. Eight of these markers (~28%) phylogenetically conflict with the majority, and are designated as C1–C8 (Fig. 2). For each relevant node we note the insertion pattern count (Table 2).

**Table 2:** Trifurcation results for each of the major nodes investigated in this study, with P-values reported for the binomial probability KKSC (P_B_) test, as well as for the insertion ratio and ILS symmetry tests (when P_B_ ≤ 0.1).

	Trifurcation	Insertion pattern	Topology	KKSC (P_B_) Test	ILS Test	Insertion Ratio Test		
				P_B_	P_ILS_	Ratio Pattern	P_R50_	P_R20_
(i)	M.irma, c-Nota, Wall	2	(M.irma, Nota), Wall	0.5	—	—	—	—
		1 (C1)	(Nota, Wall), M.irma					
		X (blind)	(M.irma, Wall), Nota					
(ii)	Wall, Nota, Osph	6	(Wall, Nota), Osph	0.0625	0.125	(6,2)	0.1445	0.0012
		1 (C3)	(Osph, Nota), Wall					
		X (blind)	(Wall, Osph), Nota					
(iii)	M.irma, Nota, Osph (regardless of Wall)	8 (i) + (ii)	(M.irma, Nota), Osph	0.0039	0.0078	(8,0)	0.0039	<0.0001
		0	(Nota, Osph), M.irma					
		X (blind)	(M.irma, Osph), Nota					
(iv)	Wall, Nota, Mac/Osph	9 (ii) + (v)	Macropus paraphyly	0.0107	0.0215	(9,2)	0.0327	<0.0001
		1 (C2)	Macropus monophyly					
(v)	(Nota + Wall), Osph, Mac	3	((Nota + Wall), Osph), Mac	0.3125	—	—	—	—
		1 (C5)	((Nota + Wall), Mac), Osph					
		X (blind)	(Mac, Osph), (Nota + Wall)					
(vi)	Nota + Osph + Wall, Mac, Ony	3	((Nota + Osph + Wall) + Mac), Ony	0.125	—	—	—	—
		0	((Nota + Osph + Wall) + Ony), Mac					
		X (blind)	(M + Ony), (Nota + Osph + Wall) + M)					
(vii)	Ony, Lag, (Wall + M)	1	((Wall + M), Ony), Lag	0.5	—	—	—	—
		0	((Wall + M), Lag), Ony					
		X (blind)	(Ony, Lag), (Wall + M)					
(viii)	Lagostrophus, Thy, (Ony + Lag + Wall + M)	4	((Ony + Lag + Wall + M), Thy), Lagost	0.0625	0.1250	(4,0)	0.0625	0.0016
		0	((Ony + Lag + Wall + M), Lagost), Thy					
		X	(Thy, Lagost), (Ony + Lag + Wall + M)					

Wall = *Wallabia*; Nota = *M. (Notamacropus)*; Osph = *M. (Osphranter)*; Mac = *M. (Macropus)*; Ony = *Onychogalea*; Lag = *Lagorchestes*; Lagost = *Lagostrophus*; Thy = *Thylogale*; M = *Macropus*; c-Nota = core members of *M. (Notamacropus)*, which excludes *M. irma*.

### *Wallabia bicolor* is nested within the paraphyletic genus *Macropus*

Grouping *Wallabia* and the subgenus *M*. (*Notamacropus*), to the exclusion of *M*. (*Osphranter*) is supported by six shared retrotransposon markers (Fig. 2, Table 2ii). One single conflicting marker was found, C3 (K106) placing *Wallabia* outside of *M*. (*Notamacropus*)*/M*. (*Osphranter*). Overall, these retrotransposon markers favour *Wallabia* grouping with *M*. (*Notamacropus*), [6 1 X]. Our statistical testing provides P_B_ = 0.0625 and P_ILS_ = 0.125. The insertion ratio test for this *Wallabia*/*M*. (*Notamacropus*) clade (6,2) gives P_R50_ = 0.1445, P_R20_ = 0.0012 (Table 2ii).

Strong rejection of the 20% introgression/hybridisation hypothesis can be explained as follows. If 20% of the genome is shared and retained, the probability of any one marker in stem-*Notamacropus* being shared with *Wallabia* is 0.2. So among the maximum of 8 markers (6 and 2, respectively from clades ii and i in Fig. 2) that are shared by all members of *M*. (*Notamacropus*), we would expect on average, *N* = 0.2 × 8 = 1.6 to be shared by introgression with stem-*Wallabia*, and appear as support for grouping *Wallabia* with *M*. (*Notamacropus*). The remaining 6.4 markers would be expected to appear as support exclusively for *M*. (*Notamacropus*). The observed support is the reverse, with six markers supporting *Wallabia/M*. (*Notamacropus*) and only two markers supporting *M*. (*Notamacropus*).

Note that the value of *N* for gene flow from the clade including the reference genome (R) is robust to variation in retrotransposition rates among lineages. For gene flow in the opposite direction, to R, the calculation of *N* assumes equal rates of retrotransposition before the gene sharing event, between the lineage leading to R (e.g. *M*. (*Notamacropus*)) and the lineage leading to its hypothesised sister taxon (e.g. *Wallabia*). The continuity of the KERV genetic divergence profile for *Macropus eugenii* ^27^ suggests that retrotransposition rates remain similar over the short timeframes that would cover the critical periods since species divergences, in which large-scale hybridisation/introgression remains likely. However, the actual variation in retrotransposition rates for KERV-1 are not well described. Nevertheless, P_R_ values would only be overconfident in the scenario that gene flow was from *Wallabia* to *M*. (*Notamacropus*) and retrotransposition was more than twice as fast in stem-*Wallabia* than stem-*Notamacropus* (Supplementary Figure S1). Theoretically, rate differences could become more of a concern as retrotransposition patterns diverge among taxa that are more divergent, although, the probability of introgression will also decrease with divergence. Nevertheless, substantially differing mixes of retrotransposon marker families on adjacent branches might warrant additional caution or rate considerations. Otherwise, P_R_ values will typically be conservative for the given gene flow percentage. In particular, insertion rate differences along lineage R before and after introgression may reduce the power of the insertion ratio test, but will not promote false rejection of the “blind” tree. The effect of insertion rate variation is further discussed in the Supplementary Information (“Conservatism of the insertion ratio test”).

For assigning markers to the statistical tests we follow the usual practice of only including unambiguous phylogenetic patterns^18^. That is, patterns that fit one of the three trees within the trifurcation of interest without any hemiplasy or homoplasy. Ambiguous insertion patterns (or multilevel conflicts)^18^ are prevalent around the base of *M*. (*Notamacropus*), most likely due to short time intervals between divergences allowing complex patterns of ILS and introgression. Hemiplasy (or homoplasy) across multiple internal branches is required to explain ambiguous patterns, such as C1 (K106), which could place *Wallabia* with *M*. (*Notamacropus*), but excludes *Macropus irma*. Including ambiguous patterns would add five further markers (C1, C5-8) in support of grouping *Wallabia bicolor* with members of *M*. (*Notamacropus*). One additional insertion, C2 (K78) instead excludes *Wallabia* from *M*. (*Notamacropus*)*/M*. (*Osphranter*), but is also present in *M*. (*Macropus*). These ambiguous markers increase statistical support for placing *Wallabia* with *M*. (*Notamacropus*) [11 2 X], P_B_ = 0.0112, however, as Kuritzin *et al*.^18^ point out, hemiplasy across multiple internal branches contravenes the assumptions of most statistical tests for retrotransposons. Doronina *et al*.^38^ recently extended testing to the simplest (4 branch) case, but not for cases with reference genome ascertainment biases.

Looking deeper at the affinities of *Wallabia*, three additional markers are shared with both *M*. (*Notamacropus*) and *M*. (*Osphranter*), [3 1 X], P_B_ = 0.3125 (Table 2v), such that, cumulatively, nine markers unambiguously support *Wallabia bicolor* falling within a paraphyletic *Macropus* [9 1 X] P_B_ = 0.0107, P_ILS_ = 0.0215, (9,2) P_R50_ = 0.0327, P_R20_ < 0.0001 (Table 2iv). One additional marker (C5) also favours placing *Wallabia bicolor* within a paraphyletic *Macropus*, but is ambiguous on the tree, because it is *M*. (*Osphranter*) rather than *M*. (*Macropus*) that lacks the marker and is therefore excluded. Overall, the findings provide strong evidence that the monotypic swamp wallaby (*Wallabia bicolor*) is a member of *Macropus*, and not that clade’s sister taxon.

### *Macropus irma* groups with the *M. (Notamacropus)* wallabies

Placement of *Macropus irma* with the other members of *M*. (*Notamacropus*) to the exclusion of *Wallabia* is supported by two markers, with one conflicting retrotransposon marker, C1 (K106) (Fig. 2) that excludes *Macropus irma* from *M*. (*Notamacropus*)*/Wallabia*, [2 1 X], P_B_ = 0.5 (Table 2i). Thus, retrotransposon insertion markers alone provide only weak support for the monophyly of *M*. (*Notamacropus*). Setting aside the question of *Wallabia* however, and focusing only on the position of *Macropus irma* among the three *Macropus* subgenera, a total of eight (Fig. 2) unopposed markers clarify the placement of *Macropus irma* with the core members of *M*. (*Notamacropus*), [8 0 X], P_B_ = 0.0039. The ILS symmetry and insertion ratio tests strongly reject ILS and introgression/hybridization contributing the eight markers that support *Macropus irma* grouping with the core members of *M*. (*Notamacropus*) P_ILS_ = 0.0078 and (8,0) P_R50_ = 0.0039, P_R20_ < 0.0001 (Table 2iii). Here, the ILS symmetry test tells us that if the “blind” grouping of *Macropus irma* with *M*. (*Osphranter*) was the true species relationship, the 8 versus 0 asymmetry for markers supporting the two alternative, observable tree patterns is highly unlikely to result from ILS. The eight markers that support *Macropus irma* grouping with the core members of *M*. (*Notamacropus*) are also unlikely to be derived from hybridization/introgression between *Macropus irma* and *M*. (*Notamacropus*). This is because we would expect a similar or greater number of markers shared by just the core members of *M*. (*Notamacropus*), derived from the portion of the genome not shared with *Macropus irma* – however there are none (Table 2iii).

### Deeper Macropodine phylogeny

We identified conflicting phylogenetic signal at the base of *Macropus*. Three retrotransposon markers (Fig. 2, Table 2v) support grouping *M*. (*Notamacropus*), *Wallabia* and *M*. (*Osphranter*) to the exclusion of *M*. (*Macropus*). However, one marker (C5) supports grouping *M*. (*Macropus*) with *M*. (*Notamacropus*) and *Wallabia* ([3 1 X] P_B_ = 0.3125). Three markers support the monophyly of *Macropus* plus *Wallabia* [3 0 X] P_B_ = 0.125 (Table 2vi), a further two retrotransposon markers are shared between *Macropus*, *Wallabia* and *Onychogalea*, to the exclusion of *Lagorchestes*, although one of these is ambiguous, also being shared with the deeper *Thylogale*, hence [1 0 X], P_B_ = 0. 5 (Table 2vii). Finally, four markers group macropodines to the exclusion of *Lagostrophus fasciatus* (the banded hare wallaby) [4 0 X] P_B_ = 0.0625, P_ILS_ = 0.1250, (4,0) P_R50_ = 0.0625, P_R20_ = 0.0016 (Table 2viii).

### Adaptive radiation of kangaroos and wallabies

**Figure 5:**
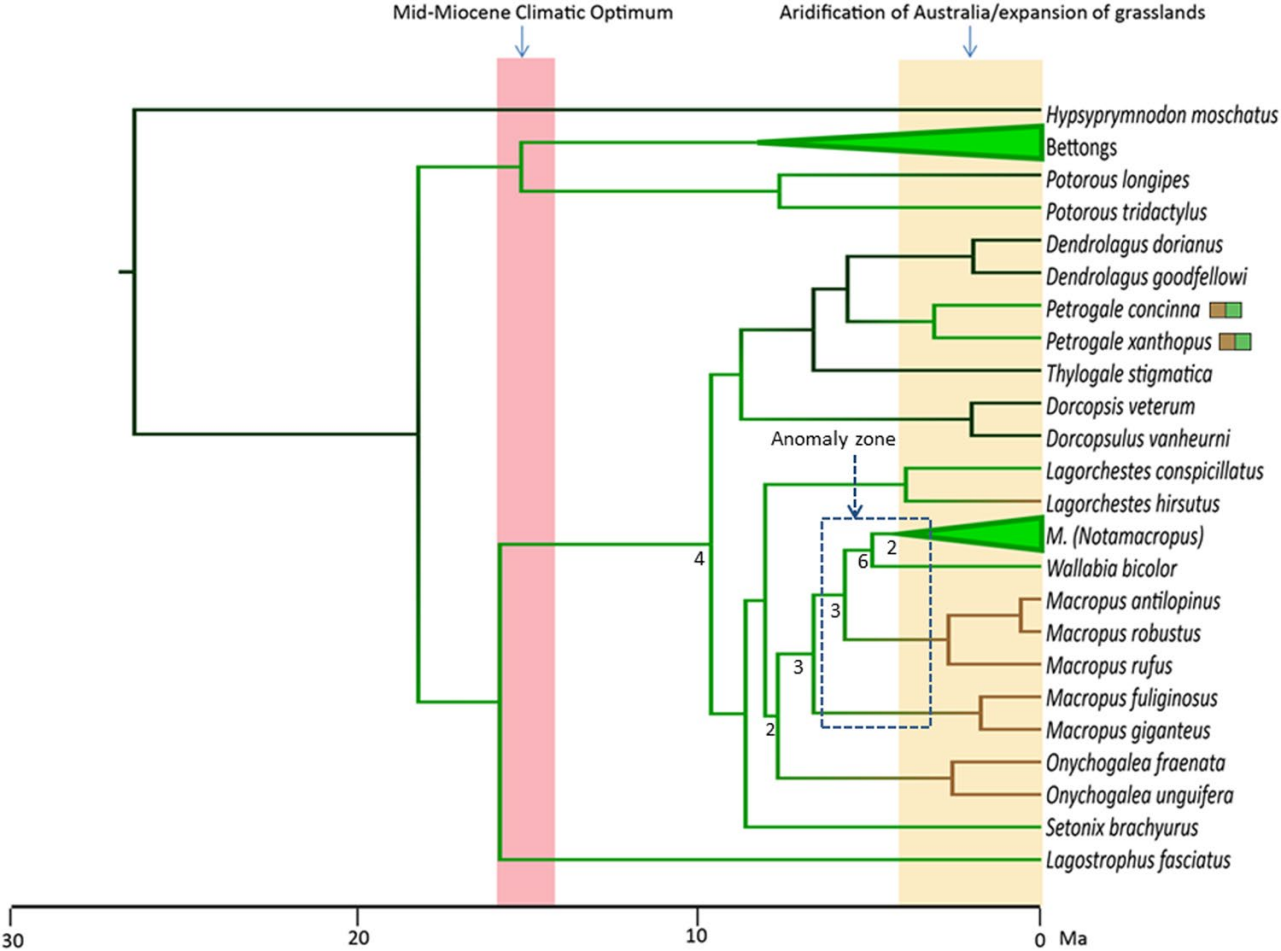
Time Calibrated Bayesian phylogeny of the Macropodidae with nodes constrained using the retrotransposon phylogeny and ancestral habitat states displayed as coloured branches. Black = Predominantly closed, wet forest; green = open canopy forest; brown = substantial range extension into more arid grasslands or other open habitats. Pink shaded window represents the mid-Miocene climatic optimum from approximately 16–15 Ma; Yellow shaded window represents the major aridification of Australia and the coincident expansion of more open habitats from ~3.6 Ma. Blue dashed-line box indicates the branches which give rise to an ‘anomaly zone’ in which substantial conflict between retrotransposon markers is found. Numbers noted below branches indicate retrotransposon marker support for each node. Small brown and green boxes next to *Petrogale* indicate that the clades represented by the included *Petrogale* taxa are polymorphic with regards to habitat, with some species having expanded into more arid grasslands, while others are more restricted to forested habitats.

To examine the implications of our phylogenetic findings based on retrotransposon markers, we inferred the timescale and habitat ancestry for the diversification of Macropodiformes. Relaxed molecular clock dating analyses of five nuclear genes for the topology in Fig. 2 estimated that the four “*Macropus*” clades, *M*. (*Macropus*), *M*. (*Osphranter*), *M*. (*Notamacropus*) and *Wallabia* successively diverged from each other over a period covering about 1.6 Ma, beginning 6.92 Ma (95% highest posterior density (HPD): 5.65–8.91 Ma) (Fig. 5).The last of these divergences, between *Wallabia* and *M*. (*Notamacropus*) was inferred at 5.33 Ma (95% HPD: 3.74–7.07 Ma). An earlier phase of diversification from about 7–9 Ma covers almost all intergeneric divergences among both potoroids and macropodines.

Ancestral habitat reconstruction employed three states: (1) predominantly closed, wet forest (e.g. rainforest), (2) open canopy forest, and (3) substantial range extension into more arid grasslands or other open habitats. Parsimony reconstruction favours a habitat expansion from open canopy forest into more open habitats prior to the divergence of *Onychogalea* and *Macropus*, well before the Late Pliocene onset of increased aridification and major grassland expansion. Under this scenario *Wallabia* and *M*. (*Notamacropus*) would require a reversal to habitats dominated by open canopy forest. Bayesian inference modelling of habitat evolution instead favours a scenario in which all expansions from open canopy forests to grassland can be dated to the Pliocene grassland expansion, and no reversals are required (Fig. 5). This Bayesian inference ancestral habitat reconstruction provides better agreement with palaeobotany^10,11^, though it still does not strongly reject an earlier transition (Supplementary Information).

### The kangaroo genome appears devoid of active LINE1 elements

We experimentally screened a region of the LINE1 ORF2 in *Macropus robustus* to further investigate the finding that each LINE-1 and SINE marker was present in all tested *Macropus* and *Wallabia* species, and thus, may lack ongoing or recent activity. From a total of 100 Sanger-sequenced clones, 99 contained deletions and/or stop codons within the analysed partial ORF2 fragment. Thus, only 1% of ORF2 sequences in our data set contained an ORF2 sequence that could be translated into amino acids. The mean within-group nucleotide distance among the 100 sequences is 0.178.

## Discussion

The first and primary statistical test for retrotransposon markers^16^ does not account for a critical ascertainment bias for which markers cannot be identified in support of clades that do not include a reference genome. In addition, Kuritzin *et al*.^18^ show that this ascertainment bias results in a loss of statistical power. Complete genome sampling for most groups remains sparse, indeed many retrotransposon studies employ a single reference genome^19–21^ and may be overstating statistical confidence and prematurely confirming or overturning DNA sequence based phylogenetic inferences.

In our study of kangaroos and wallabies, the ascertainment bias for detecting retrotransposon markers is clear – all of the identified markers fall on branches ancestral to the single reference genome, *Macropus eugenii*. This includes phylogenetically conflicting markers (hemiplasy) that support alternative groupings (Fig. 2, C1–8). However, insertion patterns for clades that exclude *Macropus eugenii* remain unobservable. Experimental approaches were employed to address this ascertainment bias. Screening of 33 introns “devoid” of retrotransposon insertions in *Macropus eugenii* did not yield any novel markers, and the approach will require many-fold more loci for effective, novel insertion discovery among kangaroos and wallabies.

In the absence of additional experimental or *in silico* evidence, three lines of reasoning were used to circumvent the single reference genome ascertainment bias. These arguments include consideration of (i) *a priori* evidence, (ii) the expectation that ILS will distribute markers that conflict with the species tree roughly symmetrically between the two alternative trees, and (iii) an insertion ratio test for whether introgression/hybridization could contribute the markers supporting the favoured “observed” tree. The latter two arguments (Figs 3 and 4) provide a basis for statistical tests that support the observed placements of *Macropus irma* and *Wallabia bicolor* with core-*Notamacropus*, and reject “blind” trees in which either *Macropus irma* or *Wallabia* would share a closer relationship with other *Macropus* subgenera (Table 2).

Overall our KKSC (P_B_), ILS and insertion ratio test results provide good agreement with Meredith *et al*.’s^27^ five nuclear gene phylogeny, indeed with the retrotransposons often lending statistically stronger support. In the one case of disagreement (retrotransposons favour *Onychogalea* instead of *Lagorchestes* as sister to *Macropus/Wallabia*), the nuclear sequence result was poorly resolved (57% ML bootstrap support), and the retrotransposon grouping agrees with morphological studies^5,7,8^. Retrotransposon support for *M*. *eugenii* grouping with *M*. *parma* instead of with *M*. *agilis* as found by Meredith *et al*.^3^ is not incongruence, but results from the latter study mislabeling *M*. *eugenii* and *M*. *agilis*.

Retrotransposon markers strongly support placing *Wallabia bicolor* within *Macropus* [9 1 X] (Fig. 2, Table 2iv) in agreement with nuclear genes^3^, and overturning *Macropus* monophyly, which has generally been favoured by morphological studies^7,8^ and by mitochondrial DNA^9,39^. For the more precise placement of *Wallabia*, our data suggest this genus is sister to the subgenus *M*. (*Notamacropus*), an arrangement that Meredith *et al*.^3^ weakly favoured, based on a five nuclear gene concatenation. Our retrotransposon markers provide stronger support for this *Wallabia/M*. (*Notamacropus*) clade (Fig. 2 and Table 2ii), although our ILS symmetry and insertion ratio tests do not reject the “blind” *Wallabia/M*. (*Osphranter*) clade at P = 0.05. *A priori* evidence strengthens the argument, because no previous molecular or morphological phylogenetic investigations favour the “blind” tree, leaving the most relevant comparison as the strong binary preference for *Wallabia/M*. (*Notamacropus*) over *M*. (*Notamacropus*)/*M*. (*Osphranter*).

Conflicting retrotransposon markers have been shown to be common when lineages diverge in rapid succession^34,35^. The present study on kangaroo and wallaby relationships is remarkable however, in the complexity of the hemiplasy. For example, four alternative insertion patterns place *Wallabia bicolor* with different groupings within *Macropus* that do not appear on the species tree (Fig. 2, C1, C5, C6/7, and C8). These provide additional support for *Macropus* paraphyly, although, for our statistical analyses we only include unambiguous markers (those without multilevel conflicts). This diversity of conflict is consistent with rapid successive divergences among the *Macropus* subgenera and within *M*. (*Notamacropus*), allowing ILS and perhaps introgression to span several internal branches on the species tree. From the origin of *Macropus* to the base of *M*. (*Notamacropus*), each of the three internal branches with conflict patterns, have estimated durations of ~0.8 Ma (Fig. 5). In contrast, branches supported by three or more insertions, and not subject to hemiplasy were longer (>1.0 Ma). Interestingly, the one conflict pattern that excludes *Wallabia* from *Macropus* (C2, Fig. 2) requires at least 1.6 Ma coalescence. The same phylogenetic placement based on mtDNA (which has lower effective population size) was suggested, based on coalescence simulations, to have arisen not by ILS, but by introgression into *Wallabia* from an extinct taxon outside of *Macropus* ^4^.

Phillips *et al*.’s^4^ coalescent simulation study also showed potential for shallower ILS for nuclear loci between *Wallabia bicolor* and the *Macropus* subgenera. In all cases the conflicting markers have the same diagnostic ERV mutations and are therefore unlikely to result from independent insertion events of different ERVs. We cannot exclude the possibility of exact deletions of ERVs, however, exact deletions are very rare in other retrotransposon studies, and comprise <0.5% in primate genomes^40^.

The placement of *Wallabia bicolor* within the genus *Macropus*, as sister to *M*. (*Notamacropus*) presents a taxonomic anomaly. Meredith *et al*.^3^ suggested subsuming *Wallabia bicolor* into the genus *Macropus*, with the creation of a new subgenus, *M*. (*Wallabia*). Another possibility is maintaining *Wallabia*, and instead elevating the three *Macropus* subgenera (*Osphranter*, *Macropus* and *Notomacropus*) to independent genera^41^. Short internal branches separating the subgenera (~0.8 million years) and the potential for hybridization, even if offspring are typically sterile^42,43^ may favour subsuming *Wallabia bicolor* into *Macropus*. Conversely, substantial behavioural and ecological differences between each of the *Macropus* subgenera and *Wallabia* argue for elevating each to the genus level. Morphological considerations are also required to guide this taxonomic decision, while resolving the affinities of *Macropus* fossils should allow more confident temporal and ecological inferences of the group’s diversification.

There are two clear *a priori* hypotheses for the placement of *Macropus irma*; the first, a close affinity with *M*. (*Notamacropus*), is based on morphology^44^ and five nuclear genes^3^. The alternative, which places *Macropus irma* with *M*. (*Osphranter*), based on mtDNA^4^ is the “blind” grouping for this retrotransposon study. Thus, the *a priori* evidence argument cannot be used to reduce the emphasis on the “blind” tree for *Macropus irma* affinities. However, support from retrotransposons alone is sufficiently strong to confidently group *Macropus irma* (with or without *Wallabia*) with the core members of *M*. (*Notamacropus*) and reject the “blind” tree ([8 0 X] Table 2iii). Combining the ILS symmetry, insertion ratio, and *a priori* evidence arguments has substantially overcome the limitations of a single reference genome being available, and lends confidence to placing *Macropus irma* and *Wallabia* as consecutive sister taxa to the core members of *M*. (*Notamacropus*).

The relationship among the three *Macropus* subgenera remains unclear. Three markers group together *M*. (*Notamacropus*)*/Wallabia* and *M*. (*Osphranter*) in agreement with numerous molecular studies, including early serological studies^45^, DNA hybridization^45–47^ and nuclear genes^3^. However, two conflicting markers were found that group *M*. (*Macropus*) and *M*. (*Notmamacropus*) together, to the exclusion of *M*. (*Osphranter*). One of these markers includes *Wallabia bicolor* (Fig. 2, C5) and the other does not (C4). This hemiplasy across short internal branches over successive divergences is consistent with speciation events early in *Macropus* occurring more rapidly than the rate of allele fixation. Greater resolution from additional markers will be required or indeed the basal *Macropus* trichotomy may be unresolvable, as has been suggested for the deep divergences within placental mammals^48^ and among avian orders^49^. An additional reference genome will allow assessment of the “blind” alternative among the three subgenera, specifically the grouping of *M*. (*Macropus*) with *M*. (*Osphranter*), which is generally favoured by morphology^8^ and mtDNA^39^.

Two shared retrotransposon markers (one phylogenetically unambiguous) provide the first molecular evidence for a close relationship between *Onychogalea* and the *Macropus/Wallabia* clade (Fig. 2). This grouping has often been weakly supported by morphology, particularly dental traits that appear to have evolved for grazing^8,50^. In contrast, recent molecular analyses^3,39^ tend to favour a deeper affinity for *Onychogalea*, outside the clade containing *Lagorchestes* and *Macropus/Wallabia*, though with very weak statistical support. Confirming the placement of *Onychogalea* with *Macropus/Wallabia* will require additional markers, the ability to rule out the “blind” *Onychogalea/Lagorchestes* relationship, and resolving the phylogenetic position of the quokka (*Setonix*).

With retrotransposon markers clarifying several phylogenetic placements, and addressing a need for more robust fossil calibrations^50^, our estimate for the crown origin of *Macropus/Wallabia* of 6.92 (5.65–8.91) Ma is slightly younger and more precise than most earlier estimates^3,4^. Deeper in the tree, the crown origins of both major macropod families, Macropodidae and Potoroidae coincide with the mid-Miocene climatic optimum, about 15–16 Ma (Fig. 5) when rainforest was more widespread across Australia compared to the Late Miocene, onwards^51,52^. Our habitat reconstruction places the ancestors of both potoroids and macropodids in open canopy forest, potentially advantaging both groups of taxa as open forests expanded later in the Miocene. Open forests already existed during the Oligocene^53^, when the initial transition from rainforest to open-canopy forests is likely to have occurred among macropods (Fig. 5).

Transitions or expansions from open canopy forest habitats to more open and widespread grasslands are inferred to have occurred independently in the lineages leading to *Onychogalea*, *M*. (*Macropus*), *M*. (*Osphranter*), *Lagorchestes hirsutus*, and within *Petrogale*. Each of these transitions falls on branches that temporally match the development of Australia’s grasslands, which became widespread by the Late Pliocene^10,11^ (3.6–2.6 Ma). These inferences are also consistent with forest-dwelling being retained earlier, in the oldest known (~5–4.5 Ma)^54,55^ putative members of both *Macropus* and their close relative, *Protemnodon* ^42^.

The composition of transposable elements in the genome can vary dramatically between taxonomic groups^56^. Our transposable element screen of *Macropus* and *Wallabia* revealed little or no LINE1 activity over the last 10 million years. Instead only ERV markers were found, which are generally widespread in kangaroo and other mammalian genomes^57,58^. Our phylogenetic analysis of the ERV solo LTRs found in the *Macropus eugenii* genome shows a clustering of different clades, with the majority of young LTRs occurring within a single clade with some heterozygous markers, characteristic of recent insertion events that have not reached fixation (Supplementary Figure S2). The phylogenetic screen coupled with the LINE1 ORF2 screen suggests that LINE1 either has very low retrotranspositional activity, or may have become entirely inactivated in the kangaroo genome. Cases for LINE extinction among mammals have been proposed for megabats^59^, sigmodontine rodents^60–62^, Tasmanian devil (*Sarcophilus harrisii*)^21^, thirteen-lined squirrel (*Ictidomys tridecemlineatus*)^63^, and the spider monkey (*Ateles paniscus*)^64^. It is possible that ERV activity in the kangaroo genome may have increased due to the absence of competition from LINE1 activity, and indeed parallels have been observed in sigmodontine rodents^65^. Further screening of high quality genome assemblies will make it possible to explore the evolutionary interplay between LINE1 and ERVs in the kangaroo genome.

## Conclusions

In this study, we provide a statistical framework for accommodating the retrotransposon ascertainment bias that arises when only a single reference genome is utilized. This has implications for significance testing in studies performing retrotransposon-based phylogenetic reconstruction. For the first time, we are able to identify clades that are strongly supported and robust to the single genome ascertainment bias and we identify other clades that need to be tested with additional genome data. Retrotransposon support, for both previous and future single reference genome retrotransposon studies, should be assessed in a similar fashion to verify their conclusions. In addition, we have demonstrated experimentally that LINE1 silencing likely occurred in macropods. ERV markers provide highly significant phylogenetic support among kangaroos and wallabies, including for grouping the swamp wallaby, *Wallabia bicolor*, with the open forest wallabies of *M*. (*Notamacropus*). Deeper in the tree there was little phylogenetic conflict among markers, which most notably favour *Onychogalea* as closely related to *Macropus/Wallabia*. Ancestral habitat reconstruction reveals multiple independent ecological shifts among kangaroos into more open habitats, coinciding with the aridification of Australia over the past ~3.6 million years.

## Electronic supplementary material

Supplementary Information
