# Supplementary material for: Resolving kangaroo phylogeny and overcoming retrotransposon ascertainment bias
Source: Sci Rep. 2017 Dec 1;7:16811. doi: 10.1038/s41598-017-16148-0 (PMC5711953; doi:10.1038/s41598-017-16148-0)
Supplement: Supplementary file 1 — Supplementary Information [file 41598_2017_16148_MOESM1_ESM.pdf]

Supplementary Information

Resolving kangaroo phylogeny and overcoming retrotransposon ascertainment bias

William G. Dodt, Susanne Gallus, Matthew J. Phillips, Maria A. Nilsson

Contents

Supplementary Table S1. Primers

Supplementary Table S2. Taxon sampling

Supplementary Table S3. Presence/absence of phylogenetically informative ERVs

Supplementary Table S4. Retrotransposons and target site duplications table

Supplementary Table S5. Heterozygous test in *Macropus eugenii*

Supplementary Figure S1. Conservatism of the insertion ratio test

Supplementary Figure S2. Bayesian inference KERV phylogeny

Supplementary Figure S3. PCR gel-electrophoresis photo examples

Retrotransposon nexus matrix

Supplementary Methods

Supplementary Table S1. Primer list for phylogenetically informative markers, as well as for *Macropus eugenii* specific markers used in the heterozygous test.

	Locus	Forward (5' - 3')	Reverse (5' - 3')
Phylogenetically informative markers	01K	GAAGACCTGGTAGTCTTAAGACAGTCAGG	GATGACACAGAAATTCAGATGGAGC
	02K	GTGCACTTTAAAGTGCAGCAACAAC	GAGAAGTCATTGAAACTGGGTTAGTGG
	03K	CTCCTCCAGCCAGCAGTCCTGAC	CAGTAAGAAATGGGACCCACTGATC
	11K	CAACTGTGGTTATTTTGCATTCTTG	GATGGAGCTCAAACTCCTCCAGAGGGC
	70K	CCAGTTCGGCATTTCCATAG	GGTCCCAGGAAAATCATTTG
	71K	GTAATTCCAAAGGCTAGTCCTTC	CCTAGTCTGAGGCAGTCATG
	76K	CTCTACCAGGATTGTCCCATG	CTATGGCTAGATCTTCCAAATATGC
	78K	CTGTCTATTCTTGCAGATCCAATC	GATTGATCAGTTTCCAGTGTCTATTC
	79K	GACAAGGAGAATGACTGTAGAACTG	CACTGTTCCCTGCTCAGTC
	97K	GTTCACCTGGATGCCATG	GTTGCTTCATCATGTTTGTCTC
	99K	CAGAATCCCTTACCACCTGTGAC	GGTCAGATTGAATTAATGGTAACAATG
	100K	CATCTTTTGATGAAGTGTCTCAG	CTCTGGAATATAACAGTATGAAACTAG
	101K	GGAAGACCTTGTAGAGTATGGTAAC	CACACTGATGTTACTCTGTTCC
	102K	GGCTTTAATTACTAGGACATTC	GTAATCCTAGCTCAGGGTAAC
	104K	GTTAACTCAATGACCAAAGGAATAAC	CCTAACTTTCACCATGAGGGTC
	106K	GTATCACACAAGGTTAATAGAGATC	CCATGACATTATACTCTCACAGTC
	107K	GCTTTCCAAAAAGTCGACCAG	GACAAGCCAGAATAGATTAATTTGA
	122K	CTTGTCATTATCCTTACTCTTTCCTTC	GTGAGAAGGTCTTAAATCATGATC
	123K	CAATGTGGTGGCAAGATAGTTG	CATTCCACATCCAGTTCTCTATC
	124K	CCAATGAGTTCTGTCTTCTTATTATG	GTATCAGATTAATTCAATTCATCAAGAG
	126K	GTATGGATATCTCTAAGTGTTCATAAATG	CCTGATTGATTAAACATATGTGCTG
	127K	CAGATAGCAAGAAGCCTGG	GAGAAAGAATACATACAAATAGTGAACTCC
	130K	GATACTGCCCTGGTTTGGTAAG	GATCCTCAGTCCAGGAGTCC
	131K	CCATTATTTGGATTCTTCTGAGATTG	GTTCCAACCCACCTATTATAGCAG
	132K	CAATTCCATACAATTATAGTTAACTTTAAG	GTAGAGATGATATCTTATTAGGTCCTTG
	133K	GTAACAGATGTCAAGGCTACAGAGTTAG	CAATGCTTGTTATCTCATCCATGAG
	135K	GATCCATATGAATTACTGAATTC	GGTAAAGGTTAATCAAATTCTG
	136K	CAGTTCCTTCTTCCACTGTTAG	GTAAATTGGGATCAGATTGTG
	137K	CCTTTGGACTGAGTCCAGTGTC	CTGCCAGGAGCACAATCATC
M.eugenii specific markers (heterozygous test)	04K	GTAGACTCCTTTGCTGAGCAGAGAATGA	GTAAGGAAATTTGGCACTTGCCTTTG
	07K	CTCGAAATAATTAAAGTAATACGTGCAGG	GGAGACTAAGTTGGTTAGAGCAGGAGGC
	08K	CCTATCTAGTGGCTGATGATTTCATTC	CAAGCTCTGAGATTTGGCAGAGTCCACC
	68K	CATATAGTTCCTTATGAACTTTGCTTC	GCACAAAGTACATGTATGTGCAC
	74K	GAACAAGTCCAATCCCTTTTCTG	CAAGACTAAAAGACAGTTTACCTGAGG
	75K	CTTAATTTTCTGTCCTTGCCACTAC	CTTAGCATAGATCAGGTACAATGAAGC
	77K	CAGAACTGATTAGGAATGGAATCC	GGAAAACAGGATGTTACACATAAGAG
	84K	CTTGCTGTTATAGCTAATATTTCTGG	GCAATGAAGTAGTCCTACCAAAC
	86K	CTATCAAAGCCATCCCTTCAG	CTGAAATCAGAACCATAAGACAGAG
	88K	CTATCAGCTCTACATTGGTTGTCC	CATTACAGGTTTGCAGAAAGATC

89K	GTAGCATCATCAGACTTGTACTTTAGG	GCACAATGGATAAAACAGGAGTC
90K	GATCTATAGTAACTAACTGTGAGGACTG	GATATTCTACATGGAAAGTGGTCTATAC
92K	GCTTAGAAGCAAGTGCATTTC	CTGATACTGATGTGCTTTGGAG
93K	CCAACTGGACCTCTCCTTTG	GTAGTTTGCCACCAGAAATGG
94K	GTTTCTTCTCAATTAACAGACCTTG	CAGTTGAATGTAAGCTCCTTAAGG
95K	GTTGTGATATGGTTAGGCCATC	CCAATGCTGATTAATCACTCC
96K	CACAAAGGTTGATATCCTCTAATC	CATTGGAGCTTTGATAGAAGAT
103K	GATTGTCAGAGGGAAAGACAAG	CAGTGTTTCCTCTGGGATC
108K	GAATTAACAGGCCTCAGGAAGAC	GAAAACAGAAATCTCCAATCAGTG
109K	GACAGTCACTATTAATAGTTTTATTC	GTACCCAGAATGAGAATGAAC
110K	GTACTCATAAATGACAAGGAGATTAGC	CCTAAATACTTCTTTGGCAACTTTC
111K	GTACATTTTACTAAATATCTAGTAGCAG	GAAGTTAATGAGATGAATAGAATTC
113K	CTCCTAACTCATAGAATTACTCAAAG	GAGAATATTGTCTGGATGATATG
114K	GGAATATATTGCAGAGGTCG	GGAATGAATGTTGAGAATTG
115K	CTGTCAGTGATTTATGAGGACTG	CATAGAGCTACTACAATAAGAGAAGTAC
116K	CACTTTGAAGTCATTCACAATGAAG	GAAGGCAAAGGATCAGACATG
124K	CCAATGAGTTCTGTCTTCTTATTATG	GTATCAGATTAATTCAATTCATCAAGAG
140K	CTGAGAATGGCCAAACAG	GCCCTAAATCATTCCAGAAG
141K	GTGCTCTTTATGAAGAGTTGG	CGCACAGTTTGTAATCCTC

Supplementary Table S2. Taxon sampling for the 16 macropod species employed in the retrotransposon study.

Abbreviation	Species name	Sub-genus	Common name
Meu	*Macropus eugenii*	*Notamacropus*	tammar wallaby
Mag	*Macropus agilis*	*Notamacropus*	agile wallaby
Mpa	*Macropus parma*	*Notamacropus*	parma wallaby
Mrufo	*Macropus rufogriseus*	*Notamacropus*	red-necked wallaby
Mir	*Macropus irma*	*Notamacropus*	black-gloved wallaby
Mru	*Macropus rufus*	*Osphranter*	red kangaroo
Mro	*Macropus robustus*	*Osphranter*	common wallaroo
Mgi	*Macropus giganteus*	*Macropus*	eastern-grey kangaroo
Mfu	*Macropus fuliginosus*	*Macropus*	western-grey kangaroo
Wbi	*Wallabia bicolor*		swamp wallaby
Lco	*Lagorchestes conspicillatus*		spectacled hare-wallaby
Lhi	*Lagorchestes hirsutus*		rufous hare-wallaby
Oun	*Onychogalea unguifera*		northern nail-tail wallaby
Tth	*Thylogale thetis*		red-necked pademelon
Lfa	*Lagostrophus fasciatus*		banded hare-wallaby
Ptr	*Potorous tridactylus*		long nosed-potoroo

Supplementary Table S3. Presence/absence of phylogenetically informative ERVs. Informative markers are listed on the left using the convention of KXX for the primary retrotransposon markers and CX for the conflicting markers. Retrotransposon presence = 1; absence = 0; not amplified or sequenced = ?; amplification failure = ?*. Deletion events are represented by 'DEL' for each species. See Table S2 for species abbreviations. Dark Green shading indicates verification by sequencing; Light green shading indicates PCR verification.

		M. (Notamacropus)						*M. (Osphranter)*		*M. (Macropus)*			Outgroups				
Marker		Meu	Mag	Mpa	Mrufo	Mir	Wbi	Mru	Mro	Mgi	Mfu	Lco	Lhi	Oun	Tth	Lfa	Ptr
K01		1	1	1	1	1	1	0	0	0	0	?	0	?	?	?	?
K02		1	1	?	?*	1	1	?*	1	0	0	?	0	?	0	0	0
K03		1	1	1	1	1	1	0	0	0	0	?	0	?	?	?	?
K11		1	?	?	?	?	?	?	?	?	?	?	1	1	1	0	0
K70		1	?	?	?	?	1	?	?	?	?	?	1	1	1	0	0
K71	C6	1	0	0	?*	0	1	0	0	0	0	?	0	?	?	?	?
K76		1	?	?	?	?	1	?	1	1	?	?	0	0	?*	?*	?*
K78	C2	1	1	?	1	1	0	?*	1	1	1	?	0	0	?	?	?
K79		1	?	?	?	?	1	?	1	1	1	?	0	0	0	?*	0
K97		1	1	1	0	0	0	0	0	0	0	?	?	?	?	?	?
K99		1	1	?	1	1	1	DEL	1	0	0	?	?*	0	?	?	?
K100		1	?	?	?	?	1	?	1	1	?	?	0	1	0	0	0
K101		1	1	?	?	?	1	1	1	?*	0	?	0	?	?	?	?
K102		1	1	1	1	1	0	?	0	0	?	?	0	?	?	?	?
K104		1	?	?	?	?	1	?	1	1	?	?	1	1	1	0	0
K106	C1	1	1	1	1	0	1	?	0	0	0	?	?	?	?	?	?
K107		1	?	?	?	?	1	?	1	1	?	?	1	1	1	0	?*
K122		1	1	1	1	1	1	?	0	0	?	?	0	?	?	?	?
K123		1	1	?	1	1	1	0	?*	0	?	?	?*	?	?	?	?
K124	C7	1	0	1	0	0	1	?	0	0	?	?	0	?	?	?	?
K126	C4	1	1	1	1	1	0	0	0	1	1	?	?	0	0	?	?
K127	C8	1	0	0	0	1	1	?	0	0	?	?	?*	?	?	?	?
K130	C5	1	1	1	1	1	1	0	0	1	1	0	0	?	0	?	?
K131		1	1	1	1	1	1	?	0	0	?	?	0	?	?	?	?
K132		1	1	1	1	1	1	?	0	0	?	?	?	?	?	?	?
K133		1	1	?	?	?	1	?	1	1	1	?	0	0	0	0	?
K135		1	1	?	1	1	0	0	0	0	?	?	?	?	?	?	?
K136		1	1	?	?	1	1	?	1	1	?	?	0	1	1	?	?
K137	C3	1	?*	?	1	1	0	?	1	0	?	?	0	?	?	?	?

Supplementary Table S4. Retrotransposons and target site duplications (TSDs)**.** TSD sequences for each of the phylogenetically informative KERVs are listed, as well as the sub-family to which each informative KERV belongs. KERVs that have integrated into other TE's have been indicated. Other types of TE's present in the sequence are listed.

Marker	Conflict marker	Sub-family of informative KERV	TSD sequence	Other TE types present in sequence	KERV integrated in other TE?
K01		MERVK1C_LTR	GAAATC	CHARLIE4 (DNA transposon) - present in all species	YES (integrated in CHARLIE4)
K02		MERVK1B_LTR	CAGGAA	MIR3, ERV17_MD - present in all species	NO
K03		MERVK1B_LTR	GAAACT	MIR - present in all species	YES (integrated in MIR)
K11		MERVK1C_LTR	CACAGA	none	NO
K70		MERVK1C_LTR	TATTTC	MERVK1_I - present in all species	NO
K71	C6	MERVK1B_LTR	CTTAAG	L3 - present in all species	YES (integrated in L3)
K76		MERVK1C_LTR	GAACCC	none	NO
K78	C2	MERVK1B_LTR	ATCCTC	none	NO
K79		MERVK1B_LTR	ATAGCC	L2B_ME - present in all species	NO
K97		MERVK1B_LTR	GGTAAG	L3, MAR1_MD - present in all species	YES (integrated in L3)
K99		MERVK1C_LTR	GAAACT	MIR3 - present in all species	NO
K100		MERVK1C_LTR	GAAATC	WALLSI4 - present in all species	NO
K101		MERVK1C_LTR	GGGTAG	L2B_ME - present in all species	NO
K102		MERVK1C_LTR	GGAAAG	MIR3A_MarsA, L3_ME - present in all species	NO
K104		MERVK1C_LTR	GGGGC	CHARLIE4 (DNA transposon) - present in all species	NO
K106	C1	MERVK1C_LTR	TATGTC	L2-2 - present in all species	YES (integrated in L2)
K107		MERVK1C_LTR	TATCAG	L1-3_ME - present in *Lagostrophus* only	NO
K122		MERVK1B_LTR	AAGACT	L2-2, SINE-2_MD - present in all species	NO
K123		MERVK1B_LTR	ATTATC	MIRc - present in all species	NO
K124	C7	MERVK1B_LTR	CAACAC	BovB_Ma, RTE-3_ME	YES (integrated in RTE-3_ME)
K126	C4	MERVK1B_LTR	GATTCC	none	NO
K127	C8	MERVK1B_LTR	GGCAGG	MIR - present in all species	YES (integrated in MIR)
K130	C5	MERVK1B_LTR	CTGACC	none	NO
K131		MERVK1B_LTR	TCTAT	RTE0_Mars - present in all species	NO
K132		MERVK1B_LTR	TATCAG	LTR4_ME - present in all species	NO
K133		MERVK1B_LTR	AGTATG	WRETRO - present in all species	NO
K135		MERVK1B_LTR	CAACAC	L1_Mars1b_3end - present in all species	YES (integrated in L1)
K136		MERVK1C_LTR	TTTTGC	L2-2_ME - present in all species	NO
K137	C3	MERVK1B_LTR	GTTATT	MIR, MIR3, MIR3_MarsA - present in all species	NO

Supplementary Table S5. Heterozygous test for retrotransposon insertions specific to *Macropus eugenii*, across multiple individuals. Individuals with +/- are heterozygous for the retrotransposon insertion (two distinct bands on an agarose gel). +/+ individuals are homozygous for the retrotransposon insertion (single band on an agarose gel). Grey boxes indicate an uncertain result.

MARKER	Individual 1	Individual 2	Individual 3	Individual 4	Individual 5	Individual 6
K4	+/-	+/-	+/-	+/-	+/-	+/-
K7	+/-	+/-	+/-	+/-	+/-	+/-
K8	+/-	+/-	+/-	+/-	+/-	+/-
K68	+/+	+/+	+/+	+/+	+/+	+/+
K74	+/+	+/+	+/+	+/+	+/+	+/+
K75	+/+	+/+	+/+	+/+	+/+	+/+
K77	+/+	+/+	+/+	+/+	+/+	+/+
K84		+/-	+/-	+/+	+/+	+/+
K86	+/+	+/+	+/+	+/+	+/+	+/+
K88	+/-	+/-	+/-	+/-	+/-	+/-
K89	+/+	+/+	+/+	+/+	+/+	+/+
K90			+/+	+/+	+/+	
K92		+/+	+/+	+/+		+/+
K93	+/+	+/+	+/+	+/+	+/+	+/+
K94	+/+	+/+	+/+	+/+	+/+	+/+
K95	+/+	+/+	+/+	+/+	+/+	+/+
K96	+/+	+/+	+/+	+/+	+/+	+/+
K103	+/+	+/+	+/+	+/+	+/+	+/+
K108						+/+
K109	+/+	+/+	+/+	+/+	+/+	+/+
K110	+/+	+/+	+/+	+/+	+/+	+/+
K111	+/+	+/+	+/+	+/+	+/+	+/+
K113				+/+	+/+	+/+
K114	+/+	+/+	+/+	+/+	+/+	+/+
K115	+/+	+/+	+/+	+/+	+/+	+/+
K116	+/+	+/+	+/+	+/+	+/+	+/+
K124	+/+	+/+	+/+	+/+	+/+	+/+
K140	+/+	+/+	+/+	+/+	+/+	+/+
K141	+/+	+/+	+/+	+/+	+/+	+/+

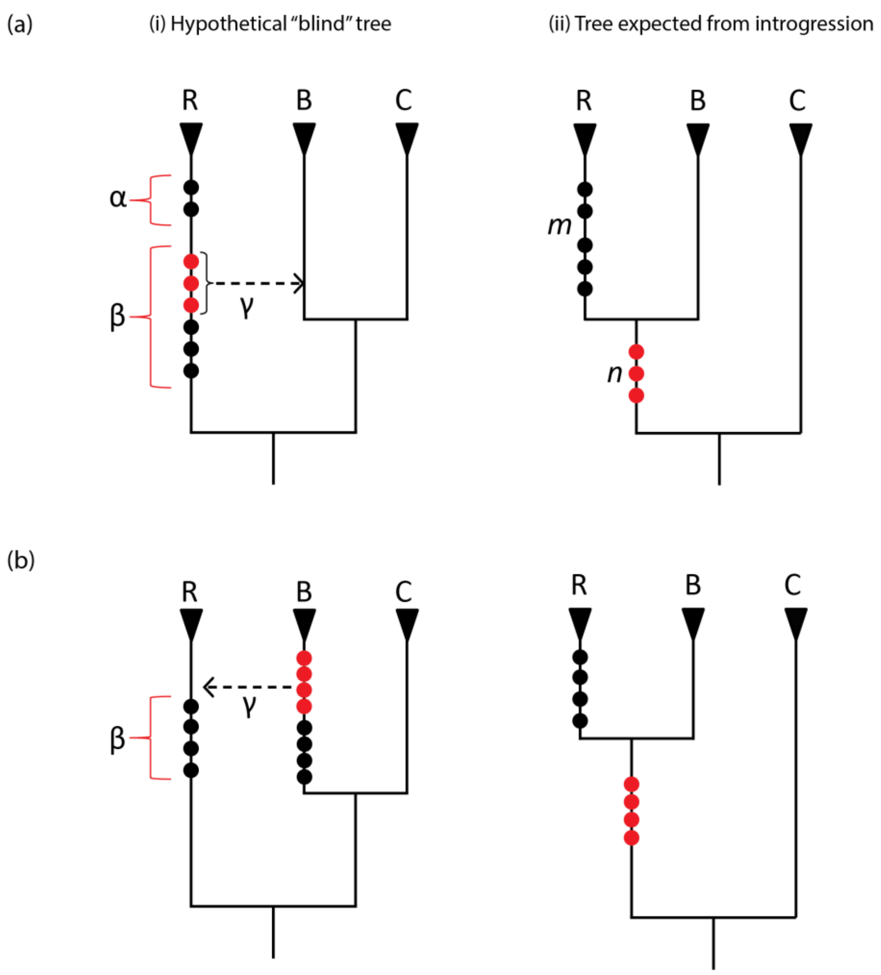

Supplementary Figure S1. Conservatism of the insertion ratio test. Hypothetical scenario illustrating the conservative nature of the 'insertion ratio argument' for accepting or rejecting the "blind" tree under the assumption of introgression/hybridization. α= insertions present after the introgression event that are specific to lineage R; β= insertions present before the introgression event in lineage R; γ= the proportion of the genome shared between the two lineages after introgression. (a) Represents introgression from the reference genome, R, into lineage B. (b) represents the reverse scenario in which introgression occurs from lineage B into the reference genome, R, when the insertion rate in B is twice that of R, and no insertions occurred after the introgression event (i.e. α=0). For the insertion ratio test to be overconfident, then $n/(n+m)$ must be greater than γ. However even in the extreme scenario of (b), the test yields a conservative outcome (i.e. $n/(n+m)$ is equal to or less than γ).

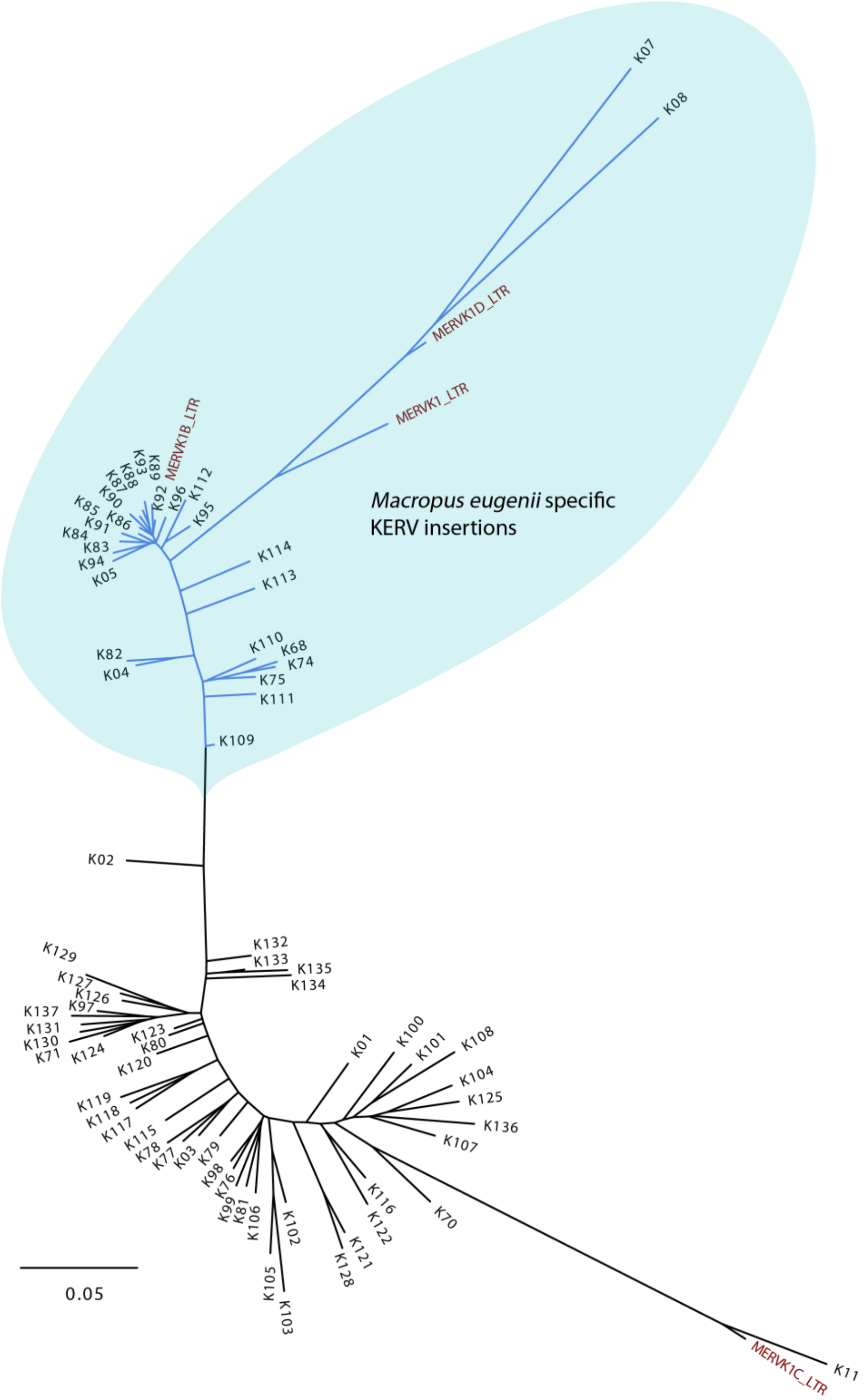

Supplementary Figure S2. Bayesian inference phylogeny, for the different sub-families of the endogenous retrovirus, KERV, based on long terminal repeats (LTRs). The blue shaded region contains the majority of the heterozygous insertions and thus represents a potentially young clade that may have arisen through a recent KERV expansion in the tammar wallaby genome. Labels coloured red indicate KERV consensus sequences obtained from Repbase and follow the naming convention for the sub-families from Repbase (eg. MERVK1C_LTR).

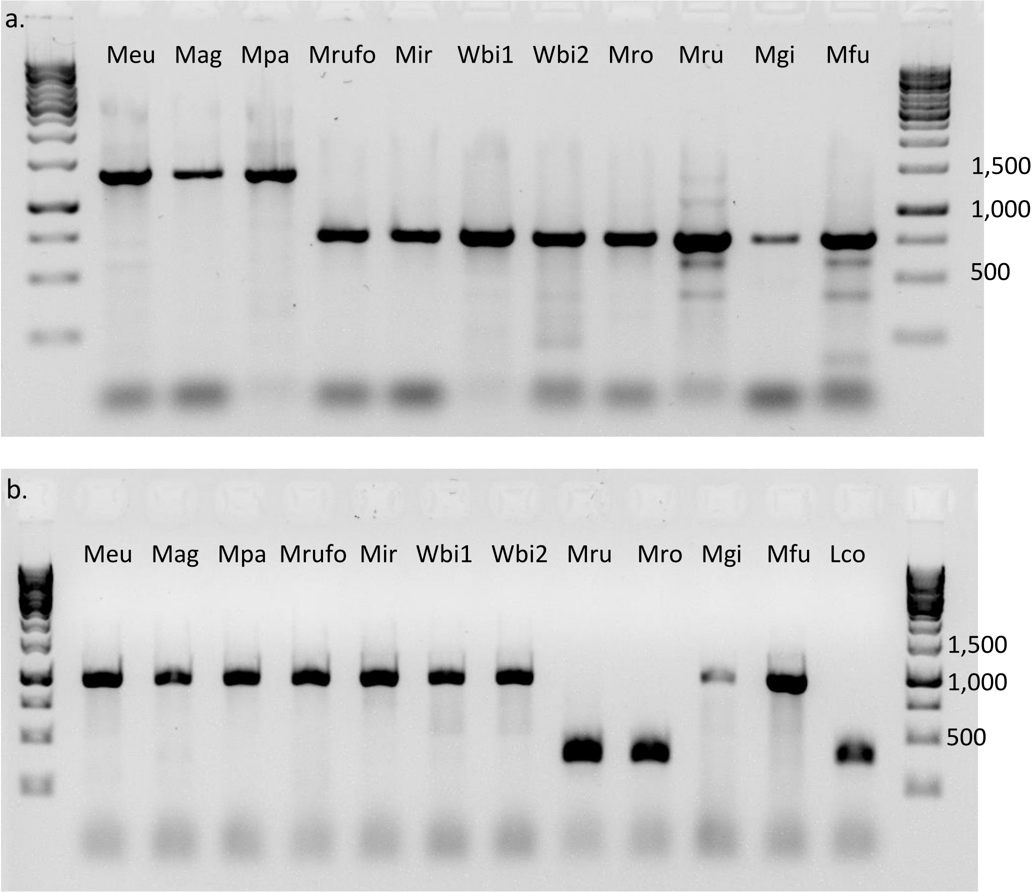

Supplementary Figure S3. PCR gel-electrophoresis photo examples. a= Marker 97; b= Marker 130; Equal amounts of PCR product were loaded and run on a 1% agarose gel. Bands with higher molecular weight (higher on photo) indicate presence of the retrotransposon, while lower molecular weight bands indicate absence. For species abbreviations see Table S2.

Retrotransposon nexus matrix

```
#NEXUS

Begin data;
      Dimensions ntax=16 nchar=29;
      Format datatype=standard symbols="01" missing=?;
Matrix
           [     1          11         21           ]
M_eugenii        1111111111 1111111111 111111111
M_agilis         111??0?1?1 1?11?1?110 10111111?
M_Parma          1?1??0???1 ???1?1?1?1 10111????
M_rufog          1?1????1?0 1??1?1?110 10111?1?1
M_irma           111??0?1?0 1??1?0?110 11111?111
W_bicolor        111?111010 1110111111 011111010
M_rufus          0?0??0???0 ??1?????0? 0?0???0??
M_robustus       010??01110 1110101?0  000001011
M_giganteus      000??01110 01?0101000 101001010
M_fuliginousus   000??0?110 0?0??0???? 1?1??1???
Lagor_con        ?????????? ?????????? ??0??????
Lagor_hir        000110000? ?0001?10?0 ??00?0?00
Onycho_ung       ???11?000? 01??1?1??? 0????0?1?
Thylogale_the    ?0?11???0? ?0??1?1??? 0?0??0?1?
Lagostr_fasc     ?0?00????? ?0??0?0??? ?????0???
Potorous_tri     ?0?00???0? ?0??0????? ?????????

;
end;

begin paup;

ctype dollo.dn: all;
out Potorous_tri;

constraints knownblind =
(1,2,3,4,5,6,(7,8),(9,10),(11,12),13,14,15,16);

Hsearch addseq=rand nreps=20 constraints=knownblind enforce=yes;
contree;

end;
```

Supplementary Methods

Taxon Sampling and species verification

Samples were obtained from animal sanctuaries, zoos, museums and from road kill specimens. In cases where road kill specimens were collected, species identification was performed by experts in the field. In addition, a number of samples utilized in this study were

already available at the Queensland University of Technology and the Senckenberg Biodiversity and Climate Research Centre (Supplementary Table S2). All recent KERV integrations were screened in a panel of six *Macropus eugenii* individuals to investigate the insertions at the population level. To verify species identifications ~500 nt of the mitochondrial control region was amplified and sequenced using published mt primers [1].

Extraction of single-copy introns and/or intergenic regions containing retrotransposons

The *Macropus eugenii* genome assembly was used to extract single-copy introns and/or intergenic regions containing retrotransposons of lengths 400 - 3,200bp, which were masked using the repeat masker software http://www.repeatmasker.org [2], using the Mammalia Repbase library. Different types of retrotransposons were selected for an initial screen. Based on a previous analysis of the *Macropus eugenii* genome the youngest kangaroo SINE was found to be WALLSI2 [3]. Therefore WALLSI2 was selected along with LINE1, as well as an endogenous retrovirus (KERV). In addition, primers for 33 introns lacking repeat elements (‘empty’ introns) were obtained from a previous study [4] and screened *in-silico* using BLAT [5]. Introns that lacked retrotransposon elements in the *Macropus eugenii* genome were subsequently screened experimentally to detect novel insertions in the non-reference species. After the initial experimental screening using species from the three *Macropus* subgenera and *Wallabia*, KERV, was the only element to exhibit activity relevant to the phylogenetic question.

The *in-silico* screen identified few KERV sequences in intronic regions, most likely due to the highly fragmented nature of the *Macropus eugenii* reference genome [6]. And so, the screen was extended to intergenic regions, as has been done in previous studies of similar evolutionary depth [7,8]. The KERV consensus sequence (MERVK1_LTR) and associated sub-family sequences (MERVK1B_LTR, MERVK1C_LTR, MERVK1D_LTR) were taken from Repbase and queried against the *Macropus eugenii* genome using BLAT [5]. Only hits with an identity score of 95-99% and length >500 nt were retained, which yielded 47,526 intergenic regions. Alignments of putative KERV LTR loci were created by extracting a region of 4 kb sequence flanking the KERV LTRs from the *Macropus eugenii* genome using samtools faidx [9].

PCR Conditions for retrotransposon screen

All PCR reactions were carried out in 12.5 µL reactions containing ~10 ng of template DNA, and VWR master-mix following the manufacturer's instructions, using touch-down PCR, decreasing the annealing temperature by 1°C over the initial ten cycles and followed by 24 cycles at the annealing temperature.

Scoring of presence and absence of insertions across the phylogeny

We followed common practices for establishing the presence and absence of KERV insertions among species to use them as phylogenetic markers (e.g. Suh et al. 2011; Meyer et al. 2012). The selected markers were initially amplified in a smaller set of taxa, with one representative from each of the four lineages, to establish the general location of the insertion in the phylogenetic tree. Following the initial information, additional species were amplified until the exact insertion of the marker was found. The amplification of 'filled sites', i.e. the presence of an insertion is generally ~400 nt larger than the 'empty site' (absence), and thus easy to distinguish using agarose gel electrophoresis (Supplementary figure S3 a,b). Sanger sequencing was used to verify the KERV insertion sequence as well as target site duplications in the taxa. The marker was amplified in relevant taxa, including species with absence (e.g. Suh et al. 2011), and the presence-absence information was scored with 1 (presence) or 0 (absence), combining the information from sequencing and agarose gel electrophoresis (e.g. Meyer et al. 2012**,** Mclain et al. 2012). For each analysed marker, sequences were produced and aligned and carefully analysed (Supplementary Data). Representatives from multiple taxa following the insertion and one or more taxa without the insertion were Sanger sequenced to verify PCR patterns for all markers.

Using representatives from among shallow monophyletic groups (e.g *M. giganteus* or *M. fuliginosus* and *M. robustus* or *M. rufus*) based on independent molecular studies, is commonly applied in phylogenetic studies based on retrotransposons (e.g Nishihara et al. 2009, Churakov et al. 2009, Suh et al. 2011). It is standard practice that markers relevant to deep questions do not require full sampling among shallow clades (e.g. Suh et al. 2011; Mclain et al. 2012; Meyer et al. 2012; Platt et al. 2015). For example, within *M. (Notamacropus)*, multiple sampling among these shallow taxa found no hemiplasy that extends back deeper than *Wallabia*. Similarly, for markers relevant to shallow questions, e.g.

within *M. (Notamacropus) and Wallabia*, no hemiplasy was revealed by multiple sampling outside *Macropus*. Without sequence verification, even a clear PCR presence/absence pattern can hide independent insertions or deletions [13]. Only one potential case of independent deletion was identified in a single taxon/marker across the 190 sequence verified taxon/marker combinations, and all markers regarding *Macropus*+*Wallabia* have at least one Sanger sequence per sub-genus.

Conservatism of the insertion ratio test for $H_{2\text{-Introgression}}$

The insertion ratio test considers the hypothesis that the "blind" tree is the species tree, and markers supporting the observable trees result from introgression/hybridization. In Figure S1, if instead of $\alpha=2$ as shown, the value of α was 0, then we can see in (a) that if $\alpha=0$, then the proportion of markers $n/(n+m)=0.5$, exactly the same as the value of gamma (the proportion of the genome shared between the reference taxon R and the non-reference taxon B). If instead $\alpha=2$, as depicted, then $n/(n+m)=0.375$, which is below the true value of gamma, and thus, makes for a conservative test for introgression explaining the apparent support for the R+B grouping. Notably, in (b) the direction of sharing is opposite, being B to R. In this extreme case, in which the rate of insertion along B is twice as fast as the rate of insertion along R, $n/(n+m)=0.5$, the true value of gamma. Even in this case the duration of $R<B$, and thus the insertion rate along B would be $>2\times R$. Thus, for the insertion ratio test to be overconfident, i.e. where $n/(n+m) >$ gamma, introgression would have to be from B to R, and with retrotransposition over twice the rate in B than R. Such a situation may become more likely as retrotransposition patterns diverge over longer divergences between taxa, although, the probability of introgression will also decrease with divergence.

As well as the potential for insertion rates to vary between sister lineages, they may also vary between successive branches along the stem lineage leading from the reference taxon. In theory it would be possible for a difference in insertion rates along a lineage to lead the insertion ratio test to falsely reject the hypothesis that the observed support derives from introgression, i.e. falsely rejecting the "blind" tree. The first relevant point here is that this null hypothesis involves insertions noted on successive branches (d and e) on the observed tree, e.g. figure 4(iii) that are in fact from the same branch, the R lineage on Figure 4 (i).

Thus, the appearance of these R lineage insertions being on two branches of the observed tree is an artefact of some being shared and some not.

Nevertheless, there is the possibility of rate variation along the R lineage itself, before and after the insertion event (insertions labelled beta and alpha on Figure 4 (i)). However, the insertion ratio test makes the conservative assumption that all of the markers d and e in Figure 4 (iii) were present at the time of the introgression event. This is equivalent to assuming that the insertion rate is zero along lineage R following the introgression event. Allowing for any positive value of that rate will only make the test more conservative. Therefore, insertion rate differences along lineage R before and after the introgression may reduce the power of the test, but will not promote false rejection of the "blind" tree.

Parsimony reconstruction with retrotransposon markers

A maximum parsimony strict consensus tree on the full retrotransposon data was inferred in PAUP 4.0b10 (Swofford 2002). Unobservable support for any grouping not including the reference taxon, *M. eugenii* prevents parsimony resolution. However, revealing the signal among the retrotransposons becomes possible by constraining undoubted groupings that do not include the reference taxon, and by placing a Dollo constraint on character state transitions. The constrained clades were the two *M. (Macropus)*, the two *M. (Osphranter)* and the two *Lagorchestes*. The reference taxon is at the tip of the tree and always state "1", the reverse situation from the standard usage of up-Dollo parsimony, where the outgroup is assumed to be "0". As such, we use down-Dollo parsimony.

Molecular dating and ancestral habitat reconstruction

Molecular dating and ancestral habitat reconstruction was carried out with MrBayes 3.2.6, and employed the five nuclear gene (Rag1, BRCA1, vWF, IRBP, ApoB) data matrix of Meredith et al. (2008) for 32 macropods, six outgroup diprotodontians and *Dromiciops*. Meredith et al. (2008) appear to have used *M. agilis for their M. eugenii* sequences. We instead employ the *M. eugenii* genome project sequences. To consider the implications of the retrotransposon findings, we initially ran a non-clock phylogenetic analysis with the groupings of *Wallabia/*M. *(Osphranter)/M. (Notamacropus)* and *Onychogalea*/"*Macropus*"

held consistent with the retrotransposon insertions tree, but without restricting the placement of taxa not included in the retrotransposon study, such as *Setonix*. Molecular dates and habitat ancestry were then co-inferred on the resulting topology. In all analyses evolutionary models were partitioned, with stationary base frequencies, the GTR substitution matrix, gamma shape, invariant sites, and relative branch lengths separately estimated. Four million mcmc generations, sampled every 5000th for two independent runs, each for one cold and two heated chains were sufficient to provide model ESS values in Tracer 1.6 well over 200 and spilt frequency standard deviations <0.01. For molecular dating the igr relaxed clock model was employed, with priors igrvarpr=exp(10) and clockratepr=lognorm(-6.0,0.6). The following fossil calibrations were used:

(1) Diprotodontia and (2) Vombatiformes, both with uniform bounds, 25.5 – 54.65 Ma. The lower bound is based on the minimum age of the oldest crown vombatiformes (and diprotodontians) among the Etadunna faunas, *Perikoala* [14,15]. The maximum bound is the maximum age of the Tingamarra fauna [16], which includes an assemblage of plesiomorphic marsupials and no putative crown diprotodontians. (3) Petauridae-Pseudocheiridae, with uniform bounds, 25.5-54.65 Ma based on *Pildra antiquus* [14,17] (4) Macropodiformes, with uniform bounds, 24.7-54.65 Ma. The maximum bound follows calibrations 1-3, however, the minimum bound is based on the earliest well established macropodoids, such as *Bulungamaya* [18] from Etadunna Faunal Zone C. (5) Macropodoidea, with truncated lognormal bounds, 17.79 – 28.5 Ma. This calibration is based on [19] but with the minimum age updated in view of new radiometric dates for *Ganguroo* at the Neville's Garden site at Riversleigh [20]. The distribution mean (23.03 Ma) is placed at the Oligo-Miocene boundary, recognizing close macropodoid crown/stem transitional forms from around this time (e.g. [21]). The maximum bound is "soft", allowing for the possibility of origins pre-dating the base of the Late Oligocene. (6) *Thylogale*-Dendrolagini, with uniform bounds, 4.36-14.22 Ma. The minimum is based on *Thylogale ignis* fossils from the Hamilton fauna [22] and the maximum bound is the maximum age of Ringtail site at Riversleigh and recognises the absence of Macropodinae from Riversleigh Faunal Zone C sites or contemporaneous sites elsewhere. For ancestral state reconstruction, primary habitat was coded (0) rainforest, (1) open canopy forest, (2) grassland based on known habitat preferences [23,24]. Most species of kangaroos and wallabies will be found in open canopy forest/woodland at least some of the time, however, the rainforest state here is defined as such, only when the majority of populations from a species/clade predominantly occupy rainforest or other Mesic closed canopy habitats. The

grassland state is distinguished by distributions extending into sparsely treed, more arid grasslands or other open habitats. These grassland specialized macropods are typically dentally specialized grazers, including *M. (Osphranter)*, *M. (Macropus)* and *Onychogalea* (see Sanson, 1989). In terms of ecology, geography and adaptation, state 1 is intermediate between states 0 and 2, and therefore this character was treated as ordered, and variable.

Phylogenetic analysis of KERV sub-families

The nucleotide sequences from the 83 experimentally screened KERV solo-LTRs were extracted and used to build an alignment. The four KERV solo-LTR consensus sequences obtained from Repbase (MERVK1_LTR, MERVK1B_LTR, MERVK1C_LTR and MERVK1D_LTR) were included in the alignment in order to identify the clades to which the experimentally screened loci belonged. The data set was aligned in MUSCLE [26], and poorly aligned regions were subsequently removed using Gblocks [27]. The resulting alignment was used to construct a Bayesian inference tree using MrBayes 3.3.6 [28] under a HKY-GAMMA model, as favoured by AIC within ModelTest 3.7 [29]. The presence/absence information from the experimental screen was combined with the phylogenetic ERV tree to gain a deeper understanding of when the different LTRs were active.

Investigation of LINE1 activity in the Macropus genome

The genome of *Macropus robustus* was experimentally screened for intact LINE1 ORF2 reading frames. In mammalian LINE1 sequences, ORF2 encodes the endonuclease and reverse transcriptase necessary to perform retrotransposition. A published protocol [30] was modified [31] to identify intact LINE1 ORF2 sequences. The primers F:CTCTTTGCAGATGATATGATG and R:ACCTARTMTATTCCACTGATG, located at position 4,060 – 4,674 of L1-1_ME (the youngest LINE1 in the kangaroo genome), were used to amplify this region from genomic DNA. The resulting 614 nt PCR product was purified and cloned into a Topo-TA vector (Invitrogen) and transformed into TOP10 cells. 100 random colonies were picked and used for colony PCRs using the primers M13F and M13R, and the resulting clones with inserts were sequenced. The sequences were screened in Geneious (Biomatters) to identify clones for which the entire sequence could be translated without stop codons or indels disrupting the reading frame.

The within group mean nucleotide distance was estimated after primer removal with MEGA6 [32]. Data generated from two species with known LINE1 retrotranspositional activity, the opossum (*Monodelphis domestica*) and human (*Homo sapiens*) were generated using the same protocol and used as controls [31].

Accession numbers

All DNA sequences were uploaded to Genbank under accession numbers:

Transposable element markers: LT598171-LT598344

LINE1 ORF2 sequences: LT598345-LT598445

References

1. Fumagalli, L., Pope, L. C., Taberlet, P. & Moritz, C. Versatile primers for the amplification of the mitochondrial DNA control region in marsupials. *Molecular Ecology* **6**, 1199-1201 (1997).
2. Smit, A., Hubley, R. & Green, P. http:// www. repeatmasker. org. *RepeatMasker Open* **3**, 1996-2004 (1996).
3. Nilsson, M. A. *et al.* Tracking marsupial evolution using archaic genomic retroposon insertions. *Plos Biol* **8**, e1000436 (2010).
4. Gallus, S. *et al.* Evolutionary histories of transposable elements in the genome of the largest living marsupial carnivore, the Tasmanian devil. *Molecular biology and evolution* **32**, 1268-1283 (2015).
5. Kent, W. J. BLAT—the BLAST-like alignment tool. *Genome Res* **12**, 656-664 (2002).
6. Renfree, M. B. *et al.* Genome sequence of an Australian kangaroo, Macropus eugenii, provides insight into the evolution of mammalian reproduction and development. *Genome biology* **12**, R81 (2011).
7. Meyer, T. J. *et al.* An Alu-based phylogeny of gibbons (Hylobatidae). *Molecular biology and evolution* **29**, 3441-3450 (2012).
8. Hormozdiari, F. *et al.* Rates and patterns of great ape retrotransposition. *Proceedings of the National Academy of Sciences* **110**, 13457-13462 (2013).
9. Li, H. *et al.* The sequence alignment/map format and SAMtools. *Bioinformatics* **25**, 2078-2079 (2009).
10. Suh, A. *et al.* Mesozoic retroposons reveal parrots as the closest living relatives of passerine birds. *Nature Communications* **2**, 443 (2011).
11. Nishihara, H., Maruyama, S. & Okada, N. Retroposon analysis and recent geological data suggest near-simultaneous divergence of the three superorders of mammals. *Proceedings of the National Academy of Sciences* **106**, 5235-5240 (2009).
12. Churakov, G. *et al.* Mosaic retroposon insertion patterns in placental mammals. *Genome Res* **19**, 868-875 (2009).
13. Schmitz, J., Ohme, M. & Zischler, H. SINE Insertions in Cladistic Analyses and the Phylogenetic Affiliations of Tarsius bancanus to Other Primates. *Genetics* **157**, 777-784 (2001).
14. Woodburne, M. O. *et al.* Land mammal biostratigraphy and magnetostratigraphy of the Etadunna Formation (late Oligocene) of South Australia. *Journal of Vertebrate Paleontology* **13**, 483-515, doi:10.1080/02724634.1994.10011527 (1993).
15. Black, K. H., Archer, M., Hand, S. J. & Godthelp, H. in *Earth and life* 983-1078 (Springer, 2012).
16. Beck, R. M., Godthelp, H., Weisbecker, V., Archer, M. & Hand, S. J. Australia's oldest marsupial fossils and their biogeographical implications. *Plos One* **3**, e1858 (2008).
17. Meredith, R. W., Mendoza, M. A., Roberts, K. K., Westerman, M. & Springer, M. S. A phylogeny and timescale for the evolution of Pseudocheiridae (Marsupialia: Diprotodontia) in Australia and New Guinea. *Journal of Mammalian Evolution* **17**, 75-99 (2010).
18. Butler, K., Travouillon, K. J., Price, G. J., Archer, M. & Hand, S. J. Cookeroo, a new genus of fossil kangaroo (Marsupialia, Macropodidae) from the Oligo-Miocene of Riversleigh, northwestern Queensland, Australia. *Journal of Vertebrate Paleontology*, e1083029 (2016).
19. Phillips, M. J. Four mammal fossil calibrations: balancing competing palaeontological and molecular considerations. *Palaeontologia Electronica* **18**, 1-16 (2015).
20. Woodhead, J. *et al.* Developing a radiometrically-dated chronologic sequence for Neogene biotic change in Australia, from the Riversleigh World Heritage Area of Queensland. *Gondwana Research* **29**, 153-167 (2016).
21. Cooke, B. N., Travouillon, K. J., Archer, M. & Hand, S. J. Ganguroo robustiter, sp. nov.(Macropodoidea, Marsupialia), a middle to early late Miocene basal macropodid from

Riversleigh World Heritage Area, Australia. *Journal of Vertebrate Paleontology* **35**, e956879 (2015).
22 Flannery, T. F. *The Macropodoidea (Marsupialia) of the early Pliocene Hamilton Local Fauna, Victoria, Australia*. (Field Museum of Natural History, 1992).
23 Strahan, R. *Mammals of Australia*. (Smithsonian Inst Pr, 1995).
24 Tyndale-Biscoe, H. Life of marsupials. (2005).
25 Sanson, G. Morphological adaptations of teeth to diets and feeding in the Macropodoidea. *Kangaroos, wallabies and rat-kangaroos* **1**, 151-168 (1989).
26 Edgar, R. C. MUSCLE: multiple sequence alignment with high accuracy and high throughput. *Nucleic acids research* **32**, 1792-1797 (2004).
27 Castresana, J. Selection of conserved blocks from multiple alignments for their use in phylogenetic analysis. *Molecular biology and evolution* **17**, 540-552 (2000).
28 Ronquist, F. & Huelsenbeck, J. P. MrBayes 3: Bayesian phylogenetic inference under mixed models. *Bioinformatics* **19**, 1572-1574 (2003).
29 Posada, D. & Crandall, K. A. Modeltest: testing the model of DNA substitution. *Bioinformatics* **14**, 817-818 (1998).
30 Cantrell, M. A., Grahn, R. A., Scott, L. & Wichman, H. A. Isolation of markers from recently transposed LINE-1 retrotransposons. *Biotechniques* **29**, 1310-1317 (2000).
31 Gallus, S., Lammers, F. & Nilsson, M. When Genomics is not Enough: Experimental Evidence for a Decrease in LINE-1 Activity During the Evolution of Australian Marsupials. *Genome Biology and Evolution*, evw159 (2016).
32 Tamura, K., Stecher, G., Peterson, D., Filipski, A. & Kumar, S. MEGA6: molecular evolutionary genetics analysis version 6.0. *Molecular biology and evolution*, mst197 (2013).
